# Disrupted alternative splicing for genes implicated in splicing and ciliogenesis causes PRPF31 retinitis pigmentosa

**DOI:** 10.1038/s41467-018-06448-y

**Published:** 2018-10-12

**Authors:** Adriana Buskin, Lili Zhu, Valeria Chichagova, Basudha Basu, Sina Mozaffari-Jovin, David Dolan, Alastair Droop, Joseph Collin, Revital Bronstein, Sudeep Mehrotra, Michael Farkas, Gerrit Hilgen, Kathryn White, Kuan-Ting Pan, Achim Treumann, Dean Hallam, Katarzyna Bialas, Git Chung, Carla Mellough, Yuchun Ding, Natalio Krasnogor, Stefan Przyborski, Simon Zwolinski, Jumana Al-Aama, Sameer Alharthi, Yaobo Xu, Gabrielle Wheway, Katarzyna Szymanska, Martin McKibbin, Chris F. Inglehearn, David J. Elliott, Susan Lindsay, Robin R. Ali, David H. Steel, Lyle Armstrong, Evelyne Sernagor, Henning Urlaub, Eric Pierce, Reinhard Lührmann, Sushma-Nagaraja Grellscheid, Colin A. Johnson, Majlinda Lako

**Affiliations:** 1 Institute of Genetic Medicine, Newcastle University, Central Parkway, Newcastle upon Tyne, NE1 3BZ UK; 2 Leeds Institute of Medical Research, University of Leeds, St James’s University Hospital, Beckett Street, Leeds, LS9 7TF UK; 3 Department of Cellular Biochemistry, Max-Planck-Institute of Biophysical Chemistry, Am Fassberg 11, Goettingen, D-37077 Germany; 4 Department of Biological Sciences, Durham University, South Road, Durham, DH1 3LE UK; 5 MRC Medical Bioinformatics Centre, University of Leeds, Clarendon Way, Leeds, LS2 9JT UK; 6 Ocular Genomics Institute, Mass Eye and Ear and Harvard Medical School, 243 Charles Street, Boston, MA 02114 USA; 7 Departments of Ophthalmology and Biochemistry, Jacobs School of Medicine and Biomedical Science, State University of New York at Buffalo, 955 Main Street, Buffalo, NY 14203-1121 USA; 8 Institute of Neuroscience, Medical School, Newcastle University, Newcastle upon Tyne, NE1 7RU UK; 9 Electron Microscopy Research Services, Medical School, Newcastle University, Newcastle upon Tyne, NE1 7RU UK; 10 Institute for Cell and Molecular Biosciences, Medical School, Newcastle University, Catherine Cookson Building, Framlington Place, Newcastle upon Tyne, NE2 4HH UK; 11 Newcastle University Protein and Proteome Analysis (NUPPA), Devonshire Building, Devonshire Terrace, Newcastle upon Tyne, NE1 7RU UK; 12 Interdisciplinary Computing and Complex Biosystems (ICOS) Research Group, School of Computing, Newcastle University, Urban Sciences Building, 1 Science Square, Newcastle Helix, Newcastle upon Tyne, NE4 5TG UK; 13 Princess Al Jawhara Al-Brahim Center of Excellence in Research of Hereditary Disorders, King Abdulaziz University, 7393 Al-Malae’b St, Jeddah, 22252 Saudi Arabia; 14 Centre for Research in Biosciences, University of the West of England, Frenchay Campus, Coldharbour Lane, Bristol, BS16 1QY UK; 15 UCL Institute of Ophthalmology, 11-43 Bath Street, London, EC1V 9EL UK; 16 Bioanalytical Mass Spectrometry Group, Max-Planck-Institute for Biophysical Chemistry, Am Fassberg 11, Goettingen, D-37077 Germany; 17 Computational Biology Unit, Department of Biological Sciences, University of Bergen, Thormohlensgt 55, Bergen, N-5008 Norway

## Abstract

Mutations in pre-mRNA processing factors (PRPFs) cause autosomal-dominant retinitis pigmentosa (RP), but it is unclear why mutations in ubiquitously expressed genes cause non-syndromic retinal disease. Here, we generate transcriptome profiles from RP11 (*PRPF31*-mutated) patient-derived retinal organoids and retinal pigment epithelium (RPE), as well as *Prpf31*^+/−^ mouse tissues, which revealed that disrupted alternative splicing occurred for specific splicing programmes. Mis-splicing of genes encoding pre-mRNA splicing proteins was limited to patient-specific retinal cells and *Prpf31*^+/−^ mouse retinae and RPE. Mis-splicing of genes implicated in ciliogenesis and cellular adhesion was associated with severe RPE defects that include disrupted apical – basal polarity, reduced trans-epithelial resistance and phagocytic capacity, and decreased cilia length and incidence. Disrupted cilia morphology also occurred in patient-derived photoreceptors, associated with progressive degeneration and cellular stress. In situ gene editing of a pathogenic mutation rescued protein expression and key cellular phenotypes in RPE and photoreceptors, providing proof of concept for future therapeutic strategies.

## Abstract (web summary)

Mutations in pre-mRNA processing factors cause autosomal dominant retinitis pigmentosa. Here the authors provide insights into the pathophysiological mechanisms underlying non-syndromic retinal disease caused by heterozygous mutations in genes encoding ubiquitously expressed splicing factors.

## Introduction

Retinitis pigmentosa (RP) is one of the most common inherited forms of retinal blindness with a prevalence of about 1 in 2500 births and more than 1 million people affected worldwide^1^. RP is characterised by progressive degeneration of the mid-peripheral retina, leading to night blindness, visual field constriction and eventual loss of visual acuity. To date, there are no effective treatments for RP and it remains a medically challenging disease. About 15% of RP are autosomal-dominant forms caused by mutations in the pre-mRNA processing factors (PRPFs) *PRPF8*, *PRPF31*, *PRPF3*, *PRPF4*, *PRPF6* and *SNRNP200*^2–11^. The PRPFs are components of the U4/U6.U5 tri-snRNP (small nuclear ribonucleoprotein) subunit of the spliceosome, the large RNP complex that catalyses pre-mRNA splicing.

Alternative pre-mRNA splicing expands the coding capacity of eukaryotic genomes by differential inclusion of exons or retention of introns in mRNA that enables a relatively small number of genes to encode a diverse proteome. High levels of splicing diversity occur in the vertebrate nervous system where it is required for neuronal development and function. Mouse rod and cone photoreceptors, for example, have a specific splicing programme that is initiated prior to the development of outer segments^12^. This specific splicing programme primarily affects transcripts encoding components of photoreceptor primary cilia and outer segments, both of which are essential for phototransduction. Functional primary cilia are also required for maturation of the retinal pigment epithelium (RPE)^13^. Collectively, these data suggest that the precise regulation of splicing programmes for photoreceptor-specific transcripts, including those that are involved in ciliogenesis, are essential for retinal development.

Mutations in PRPFs affect the stoichiometry and kinetics of spliceosome assembly^14,15^, resulting in either transcriptional dysregulation of genes required for retinal function^16^ or mis-folding and aggregation of mutant PRPF proteins that trigger apoptosis in photoreceptors^16,17^. However, the disease mechanisms for PRPFs-related RP remain unclear and it is uncertain whether RPE or photoreceptors are the primary affected tissue. Paradoxically, PRPFs are ubiquitously expressed^10^, but mutations only cause retinal-specific degeneration, raising the question of why retinal cells are more susceptible to deficiencies in these splicing factors. Furthermore, PRPF animal models either do not recapitulate the human RP phenotype^18,19^, or only manifest late-onset RPE defects^20,21^.

Since human induced pluripotent stem cells (iPSCs) can be differentiated into RPE and photoreceptors^22–24^, we developed patient-derived retinal cells as unique physiologically relevant disease models in order to gain new insights into the molecular pathogenesis of splicing factor RP. We generated RPE and three-dimensional (3D) retinal organoids from iPSCs derived from four RP11 patients with variable clinical severity caused by two different *PRPF31* deletion mutations. Large-scale transcriptome analyses identified mis-splicing of cell type and patient-specific target genes affected by *PRPF31* mutations, providing unprecedented molecular characterisation of splicing-factor RP clinical phenotypes. CRISPR/Cas9 correction of a *PRPF31* mutation in cells derived from an RP11 patient with very severe RP, resulted in the rescue of molecular and cellular phenotypes, providing proof-of-concept evidence for the effectiveness of in situ gene correction.

## Results

### Derivation and characterisation of RP11-iPSCs

We ascertained three related RP type 11 patients with a *PRPF31* c.1115_1125del11 heterozygous mutation with variable phenotypic expression and one patient with severe RP with a *PRPF31* c.522_527+10del heterozygous mutation (Supplementary Data 1). Disease severity was determined according to fundus examination, visual field and visual acuity, and took account of the age at the time of examination (Supplementary Data 1). Hereafter, all patients and derived cells are referred to as RP11 accompanied by M (moderate), S (severe) and VS (very severe). Three unaffected controls are referred to as WT1 (wild type), WT2 and WT3 (Supplementary Data 1)^25,26^. Dermal skin fibroblasts were reprogrammed to iPSCs using a non-integrative RNA-based Sendai virus (Supplementary Figure 1A). All RP11-iPSCs harboured the mutation identified in fibroblast samples (Supplementary Figure 1B–E), expressed pluripotency markers (Supplementary Figure 2A–B), were free of transgenes (Supplementary Figure 2C), were genetically identical to parent fibroblasts (Supplementary Figure 2D) and clear of any genomic abnormalities (Supplementary Figure 2E). Both patient-specific and control iPSCs were able to differentiate into cells belonging to all three germ layers in vitro (Supplementary Figure 3A) and in vivo (Supplementary Figure 3B).

### RP11-RPE have functional and ultrastructural abnormalities

**Fig. 1:**
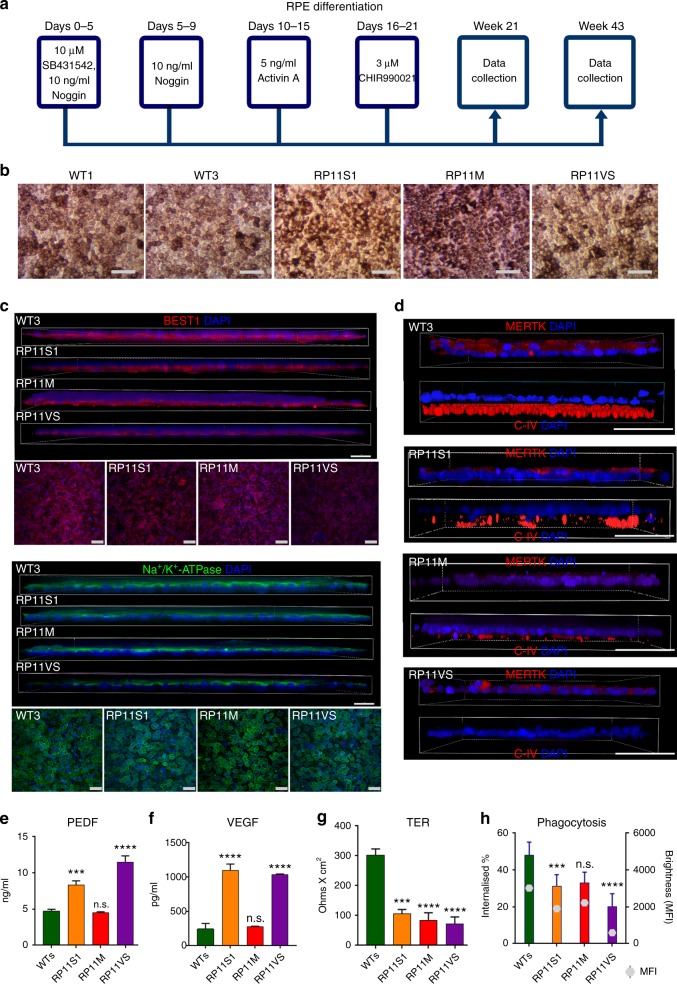
Characterisation of RP11 - RPE cells revealed polarity and functional defects. **a** Schematic of RPE differentiation timeline; **b** Bright-field images of iPSC-derived RPE: representative examples from at least ten independent experiments, scale bar 100 μm; **c** Immunostaining for basolateral markers BEST1 and Na^+^/K^+^-ATPase: representative images from three independent experiments, scale bar 50 μm; **d** Correct basolateral distribution of collagen IV (C-IV) and apical MERTK in unaffected control (WT3) but not RP11 RPE cells: representative images from three independent experiments, scale bar 50 μm; **e**, **f** ELISA assays for apical and basal secretion of PEDF and VEGF, respectively, in control and RP11 - RPE cells; **g** Trans-epithelial resistance measurements revealed a significant difference between patient and RP11 - RPE cells; **h** Reduced phagocytic capacity in RP11 - RPE cells. Statistical significance is calculated for MFI (mean fluorescence intensity) values. **e**–**h** Data shown as mean ± SEM, *n* = 3. Statistical significance of pairwise comparisons is indicated by n.s.: not significant; ***p < 0.001; ****p < 0.0001 (Student’s paired *t* test). **b**–**h** Data obtained from RPE at week 21 of differentiation

Control and RP11-iPSCs were differentiated into RPE cells using an established differentiation protocol (Fig. 1a, b). Control and RP11-iPSC-RPE showed a similar expression of the apical RPE marker Na^+^/K^+^-ATPase, but expression of the basolateral marker BEST1 was reduced in the severe (S) and very severe (VS) RP11 patients (Fig. 1c). Polarised cells in control RPE monolayers expressed MERTK in the apical layer and collagen IV in the basal layer, whereas RP11-RPE had reduced expression of both markers (Fig. 1d). Cytokine secretion assays revealed a significantly higher apical pigment epithelium-derived factor (PEDF) and basal vascular endothelial growth factor (VEGF) expression in the severe and very severe RP11 patients in comparison to control RPE (Fig. 1e, f). RPE cells produce very high levels of PEDF and polarised secretion is associated with their maturation^27–29^. Furthermore, PEDF has been shown to activate cone-specific expression and decrease rod numbers^30^. Elevated levels of such an important cytokine could therefore impair RPE polarity, with further functional consequences for rod survival. VEGF has also shown to be important for the survival of Müller cells and photoreceptors, in addition to its role in vasculogenesis^31^, and although no neovascularisation is observed in RP11 patients, dysregulated VEGF expression from RPE could have important consequences for retinal function. RP11-RPE also had an impaired ability to form a tight epithelial barrier as measured by trans-epithelial resistance (TER) assay (Fig. 1g). Furthermore, RP11-RPE derived from the two patients with severe (S) and very severe (VS) phenotypes had reduced functional ability to phagocytose rod outer segments (Fig. 1h), corroborating previous results following *PRPF31* knockdown in the ARPE-19 cell line^21^. At weeks 21 and 43 of differentiation, transmission electron microscopy (TEM) analyses revealed apical microvilli and melanosomes in control RPE, in contrast to RP11-RPE that displayed shorter and fewer microvilli, and contained large basal deposits underneath the RPE (Supplementary Figure 4). Collectively, these data indicate a loss of apical – basal polarity in patient-derived RP11-RPE.

### RP11-photoreceptors have progressive degenerative features

**Fig. 2:**
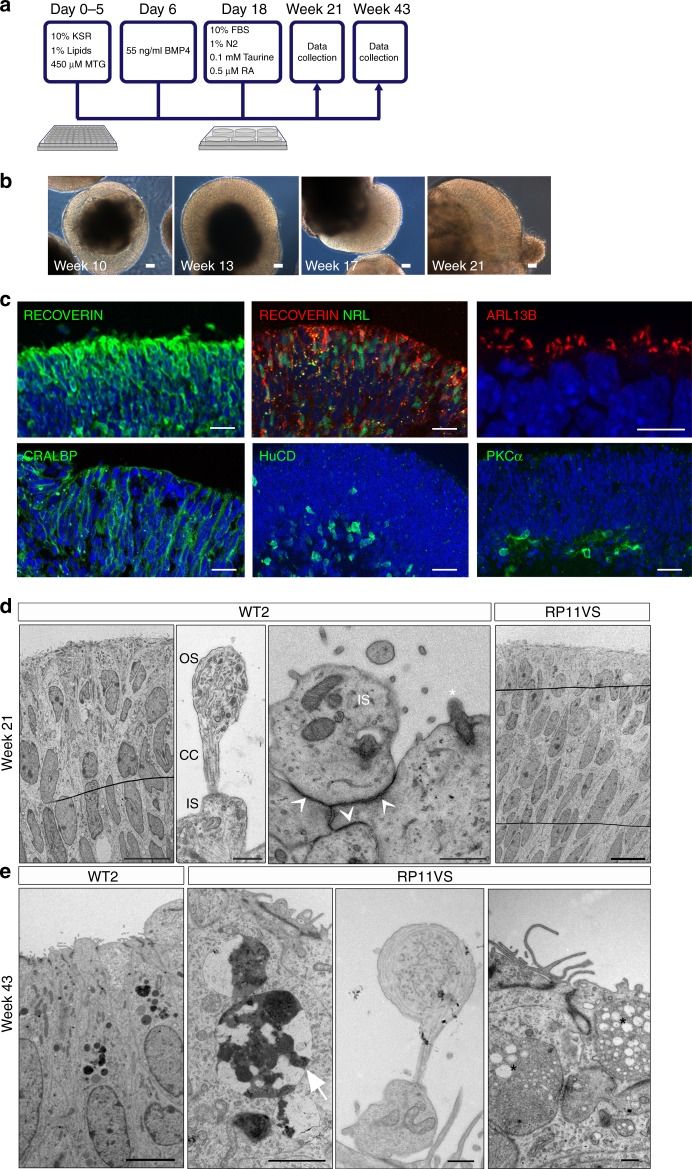
Generation of retinal organoids following long-term suspension culture. **a** Schematic representation of iPSC differentiation to retinal organoids; **b** Bright-field images showing development of retinal neuroepithelium over time, scale bar 50 μm; **c** Immunostaining of retinal organoids showing the expression of cell-specific markers; **b**–**c** representative examples from iPSC-derived retinal organoids from RP11S2 patient are shown, scale bar 25 μm apart from ARL13B, where scale bar is 10 μm; **d** TEM revealed the presence of outer limiting-like membrane (white arrows), inner segments (IS), connecting cilia (CC) and developing outer segments (OS) in retinal organoids after 21 weeks in culture, top panel scale bars: 10 μm, 500 nm, 500 nm, 10 μm, bottom panel scale bars: 5 μm, 2 μm, 500 nm, 500 nm; **e** At 43 weeks in culture, TEM showed that patient photoreceptors contained apoptotic nuclei with electron dense structures of condensed chromatin (white arrow) and stress vacuoles (black stars). **d**, **e** Representative examples of three independent experiments

We differentiated control and RP11-iPSCs into 3D retinal organoids (Fig. 2a), using an established method^24^. Bright-phase neuroepithelium developed on the apical side of retinal organoids derived from RP11 patients and controls (Fig. 2b and Supplementary Figure 5A). By week 21, retinal organoids derived from RP11 patients and controls had a well-developed apical layer packed with photoreceptors (expressing RECOVERIN) with connecting cilia (expressing ARL13B; Fig. 2c), some of which also expressed NRL indicating a rod precursor phenotype. Müller glia, ganglion and bipolar cells were also present, indicated by CRALBP, HuC/D and PKCα expression (Fig. 2c). TEM of retinal organoids at week 21 revealed the presence of outer limiting-like membrane, inner segments, connecting cilia and developing outer segments in photoreceptors residing in the apical layer of retinal organoids (Fig. 2d). There were striking morphological differences between control and patient-derived cells. RP11 - retinal organoids had a 150% increase in cells with apoptotic nuclei compared to the controls (a total of 50 TEM sections analysed). In addition, unlike WT cells, RP11 cells had 'stress vacuoles' (17% of patient TEM sections). At week 43, TEM revealed the continuing presence of apoptotic nuclei and stress vacuoles in RP11 patient-derived photoreceptors, suggesting 'adaptive survival' in response to environmental or oxidative stress (Fig. 2e).

At week 21 of differentiation, the 3D retinae were flattened down on multi-electrode arrays (MEAs) with the presumed ganglion cell layer facing down on the electrodes, in order to record action potentials generated by these cells. Measuring activity in retinal ganglion cells (RGCs) reflects the global network function of the organoid, similar to the retina, since RGCs carry the output signal to central visual areas in the form of spike trains. Control and RP11-retinal organoids had no difference in response to 8-Br-cGMP (a membrane permeable analogue of cGMP), indicating that phototransduction responses, specifically Na^+^ influx similar to the inward dark current, were intact in photoreceptors (Supplementary Figure 5B, C & F). Control retinal organoids responded to the addition of the neurotransmitter GABA with an increased firing rate, but this response was significantly reduced for those derived from the very severe RP11 patient (Supplementary Figure 5D, E & G). GABA signalling emerges during very early development, and at that time it is depolarising and can induce spiking. Reduced responses to GABA therefore indicate the impairment of emerging functional neural networks in RP11 patients.

### Impaired pre-mRNA splicing in RP11 RPE and retinal organoids

**Fig. 3:**
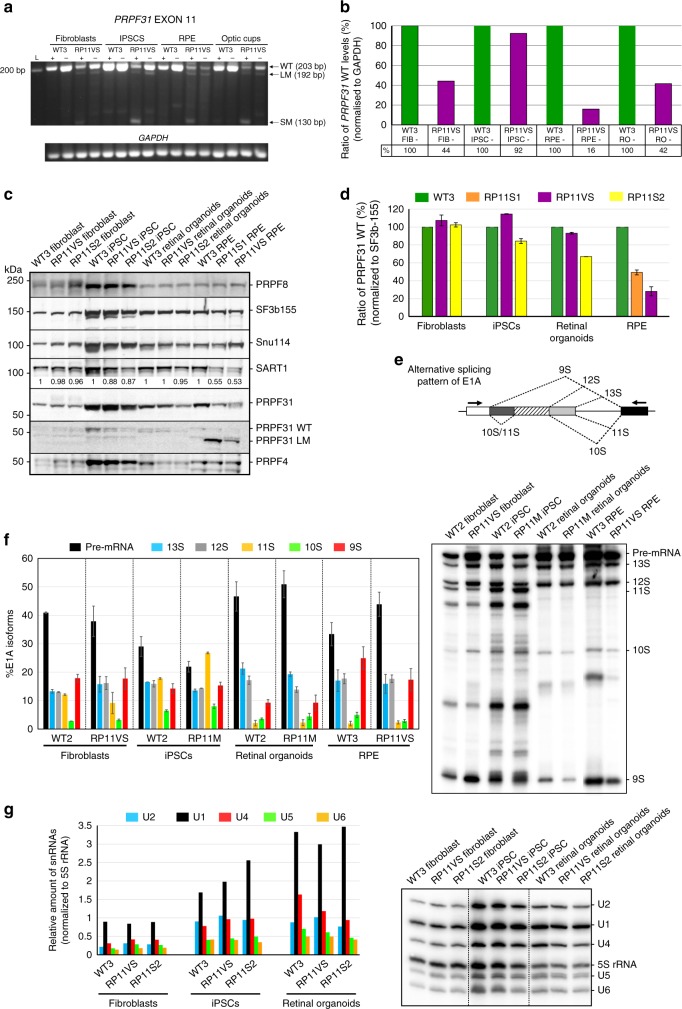
PRPF31 expression in patient-specific cells and effects on pre-mRNA splicing. **a** Gel electrophoresis showing the presence of a long mutant transcript (LM) isoform for the exon 11 deletion in patient-specific cells. The short mutant (SM) isoform is present only upon inhibition of NMD with puromycin (indicated by + ); **b** The bar graph shows wild-type *PRPF31* mRNA in patient cells relative to controls from **a**, **b**. Data are representative of at least three independent repeats, RO retinal organoids; **c** Wild-type PRPF31 is significantly reduced in patient RPE cells and less notably in retinal organoids. The LM form and reduced SART1 is observed only in the patient RPE cells; **d** The bar graph shows wild-type PRPF31 levels in patient cells relative to normal cells quantified from **c**, *n* = 3; **e**, **f** Patient RPE cells and retinal organoids exhibit a notable defect in the alternative splicing of E1A minigene reporter. Schematic representation of alternative splice variants of the E1A reporter (**e**) and denaturing PAGE and autoradiography using a phosphoimager (**f**), *n* = 3; **g** Northern blot analysis showing the level of snRNAs in various normal and patient cells. Total RNA was isolated from each sample and snRNA levels were analysed by denaturing PAGE followed by Northern blotting using probes against U1, U2, U4, U5, U6 and 5S rRNA (top). The levels of snRNAs were quantified and normalised to the amount of 5S rRNA (bottom), *n* = 2. All error bars represent SEM

To better understand the impact of *PRPF31* mutations, we performed semi-quantitative RT-PCR and western blot analysis of PRPF31 expression in primary fibroblasts, iPSCs and iPSC-derived RPE and retinal organoids. We observed and confirmed by Sanger sequencing the presence of nonsense-mediated decay (NMD)-insensitive long mutant (LM) and NMD-sensitive short mutant (SM) transcripts only in cells derived from RP11 patients with the c.1115_1125del11 mutation, but not in the control cells (Fig. 3a). *PRPF31* expression levels were decreased more significantly in RP11-RPE cells (Fig. 3b), and this was further confirmed by western blot analysis (using an anti-PRPF31 C terminus antibody) (Fig. 3c, d). Interestingly, the PRPF31 LM isoform (detected by using an anti-PRPF31 N terminus antibody) was expressed only in RP11-RPE (Fig. 3c). Furthermore, RP11-RPE showed a substantial downregulation of SART1, a U5 snRNP protein important for the formation of the pre-catalytic spliceosomal B complex, but no changes in the expression of the U5 protein PRPF8 or the U4/U6 protein PRPF4 were observed (Fig. 3c).

To test if splicing efficiency was altered in patient-specific cells, we performed splicing assays^32,33^ following lentiviral transduction of an E1A minigene reporter with multiple 5′-splice sites. This can be alternatively spliced into at least five mRNAs (sizes 13S, 12S, 11S, 10S and 9S; Fig. 3e). Both RP11-RPE and retinal organoids had impaired alternative splicing of the E1A reporter, indicated by the accumulation of pre-mRNA and the decrease in the 9S and 10S isoforms in RPE and the 12S isoform in retinal organoids (Fig. 3f) compared to unaffected control and patient-specific RP11 fibroblasts and RP11-iPSCs. There were no differences in tri-snRNP stability for RP11 and control iPSCs, as determined by sedimentation of nuclear extracts on density gradients followed by detection of snRNAs (Supplementary Figure 6). However, RP11 retinal organoids had decreased expression of U4 snRNA (Fig. 3g) compared to controls, suggesting a likely reduced function of the U2-dependent spliceosome.

### Disrupted splicing in cellular adhesion and cilia genes

To identify differences in transcription and splicing profiles between RP11 patients and unaffected control cells, we next performed large-scale transcriptome analyses in primary dermal fibroblasts, iPSCs, RPE and retinal organoids as biological triplicates from all subjects (Supplementary Data 2). We identified differentially expressed transcripts by using DESeq2 (Supplementary Data 2; threshold value *p*_adj_ < 0.05). Fewer differentially expressed genes were identified in iPSCs (*n* = 163) and RPE (*n* = 59) group comparisons, in contrast to fibroblasts (*n* = 1395) and retinal organoids (*n* = 1367). The most significant differentially expressed genes in RP11 retinal organoids (Supplementary Data 2) were enriched for Gene Ontology (GO) categories related to actin cytoskeleton, ciliary membrane, primary cilium, photoreceptor inner and outer segments, axon terminals and phototransduction (Supplementary Data 3). In RP11 fibroblasts, significant differentially expressed genes were enriched for lysosome and endosomal processes, focal adhesion, cell-substrate junctions and extracellular matrix organisation. There were no notable-enriched pathways in RP11-iPSCs and RP11-RPE.

**Fig. 4:**
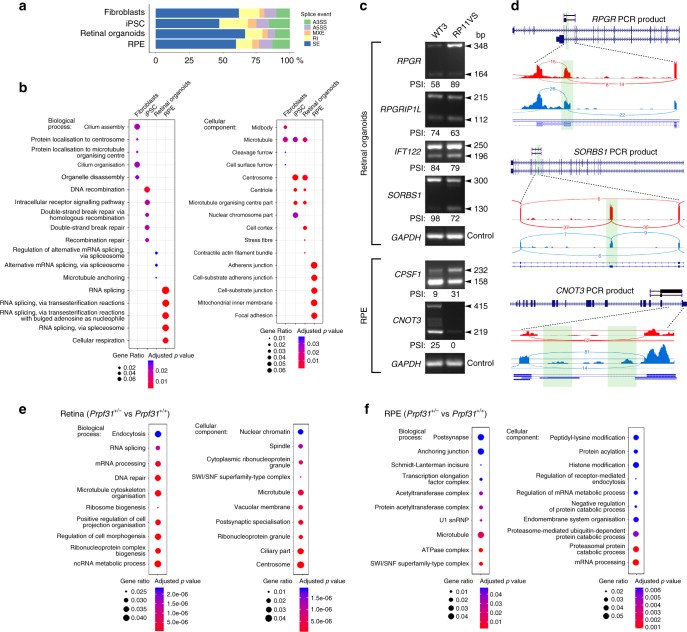
RNA-seq analysis of alternative splicing in fibroblasts, iPSC, RPE, retinal organoids and *Prpf31*^+/−^ retina. **a** rMATS analysis showing that RP11 - RPE have the highest percentage of transcripts containing retained introns (RI) and alternative 3′ splice sites (A3SS); **b** Gene Ontology enrichment analysis showing biological and cellular processes affected by alternative splicing, respectively, in human cells; **c** Gel electrophoresis of RT-PCR for the indicated genes in RPE and retinal organoids derived from patient RP11VS and unaffected control WT3. Sizes (in bp) for major and minor isoforms (arrowheads), and percentage-spliced-in (PSI) values, are indicated; **d** Sashimi plots for the indicated genes for validation of alternative splicing events in RPE and retinal organoids derived from RP11 patients (blue) and unaffected controls (red). Data are representative of at least three independent experiments. Green highlights in Sashimi plots indicate alternative splicing events with the number of junction reads indicated for each event; **e**, **f** Gene Ontology enrichment analysis showing biological and cellular processes affected by alternative splicing, respectively, in mouse *Prpf31*^+/−^ retinae and RPE. Data are representative of at least three independent experiments

Since RP11-RPE and retinal organoids had impaired pre-mRNA splicing (Fig. 3f, g), we next analysed transcripts in all four cell types for differential exon usage (skipped exons, retained introns, alternative 5′ and 3′ splice sites, and mutually exclusive exons; Supplementary Data 4) using rMATS software (threshold values *p*_adj_ < 0.05 and inclusion difference >5%). Differential exon usage analyses revealed that RP11-RPE had the highest level of transcripts with retained introns and alternative 3′ splice sites (Fig. 4a and Supplementary Data 4). GO enrichment analysis of biological processes for each cell type (Fig. 4b) showed that RP11 fibroblasts had significant differential exon usage for transcripts implicated in cilium formation categories (cilium assembly, cilium organisation, microtubule organising centre and centrosome; Supplementary Data 5). This suggests that PRPF31 has a role in fibroblast ciliogenesis and corroborates our previously published data on decreased cilia length and incidence in RP11 fibroblasts^34^. In iPSCs, enriched GO biological processes included DNA recombination and DNA double-strand break repair, whereas enriched cellular components identified the centrosome, centriole and microtubule organising centre (Fig. 4b). RP11-RPE were enriched in genes implicated in cells-to-substrate adherens junctions and focal adhesions, and mitochondrial inner membrane, whereas RP11 retinal organoids were enriched for centriole and microtubule organisation (Fig. 4b and Supplementary Data 5).

Importantly, in both RP11-RPE and retinal organoids, the most significantly enriched GO biological process was pre-mRNA and alternative mRNA splicing via the spliceosome (Supplementary Data 5 and Fig. 4b), consistent with our observations (Fig. 3f, g) that *PRPF31* mutations lead to impaired pre-mRNA splicing of key components involved in the splicing process itself. Specific human transcripts included those implicated in spliceosome assembly (e.g., *SF1, SART1/Snu66* and *DDX5*), formation of the U4/U6 snRNP (*LSM2*), 3′-end processing of pre-mRNAs (*CPSF1* and *U2AF1L4*), association of U2 snRNP with pre-mRNA (*DDX39B* and *PTPB1*) and 5′-splice site selection (*LUCL7*). To validate some of these findings, we performed RT-PCR experiments in RPE and retinal organoids derived from RP11 and control iPSC samples (Fig. 4c). For this validation we selected key genes involved in cilia formation and/or outer segments of photoreceptors (*RPGR*, *RPGRIP1L* and *CNOT3*), intraflagellar transport (*IFT122*), actin filament organisation, centrosome and focal adhesion (*SORBS1*) and pre-mRNA 3′-end processing (*CPSF1*). As predicted by the rMATS analysis, the RP11 RPE showed a significant change in alternative splicing of *CPSF1* and *CNOT3*, while RP11 retinal organoids showed alternative splicing for *RPGR*, *RPGRIP1L*, *IFT122* and *SORBS1* (Fig. 4d). These in vitro data were strongly corroborated by differential exon usage analyses of *Prpf31*^+/−^ mouse retinae and RPE^20,21^. The most significantly enriched GO processes and categories in *Prpf31*^+/−^ mouse mutant compared to wild-type control retinae were for RNA splicing, mRNA processing and ribonucleoprotein complex biogenesis, as well as microtubules, cilia and centrosomes (Fig. 4e). Similarly, *Prpf31*^+/−^ mouse mutant compared to wild-type control RPE were enriched for genes involved in mRNA processing and microtubules (Fig. 4f). These data suggest that disrupted alternative splicing programmes in RP11 result in exacerbation of splicing deficiencies, in turn disrupting specific biological processes that cause the unique cellular phenotypes observed in RP.

### RP11 - RPE and photoreceptors show ciliary abnormalities

To analyse the role of PRPF31 in cilia formation and function, we first determined the extent of co-localisation of PRPF31 with an snRNP-specific marker (Y12, Supplementary Figure 7A) and a cilia-specific marker (ARL13B, Supplementary Figure 7B) in fibroblasts, iPSCs, RPE and photoreceptors. PRPF31 co-localised with both snRNPs and ARL13B for all patient cell types and controls, confirming PRPF31 localisation in both splicing complexes and cilia.

**Fig. 5:**
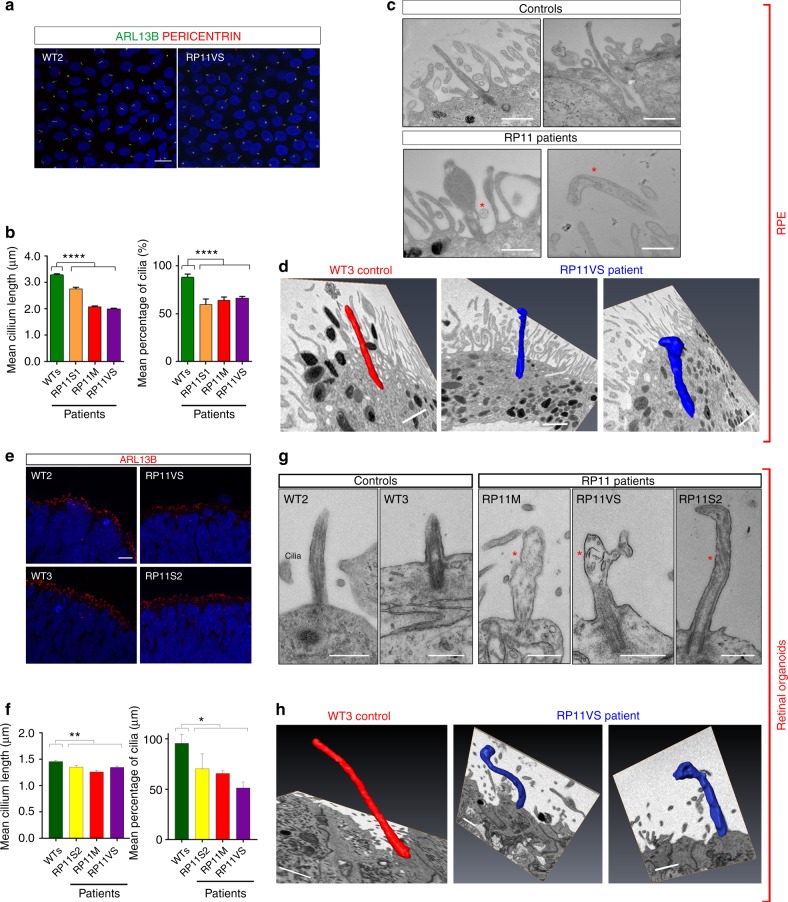
RP11 - RPE cells and photoreceptors have defective ciliogenesis and cilia morphology. **a** Immunostaining of RPE with cilia markers ARL13B (green) and pericentrin (red), with representative images shown from *n* = 3 independent experiments, scale bar 10 μm; **b** Quantification of cilia length and incidence showing significant reduction across both parameters in RP11 patients compared to the controls; **c**, **d** 2D TEM and 3D SBFSEM images showing shorter cilia in RP11 - RPE cells, with abnormal bulbous morphology, with representative images shown from *n* = 3 independent experiments, scale bar 500 nm (**c**), 1 μm (**d**); **e** Immunostaining of photoreceptors with cilia marker ARL13B (red), with representative images shown from *n* = 3 independent experiments; **f** Quantification of cilia length and frequency in photoreceptors showing significant reduction in RP11 patients compared to the controls; **g**, **h** 2D TEM and 3D SFBSEM images showing shorter cilia in patient-derived photoreceptors, with abnormal bulbous morphology, with representative images shown from *n* = 3 independent experiments, scale bar 500 nm (**g**), 1 μm (**h**). **b**, **d**, **f**, **h** Data shown as mean ± SEM, *n* = 3. Statistical significance of the indicated comparisons is indicated by n.s. not significant; ***p < 0.001; ****p < 0.0001 (one-way ANOVA test with Dunnett’s post hoc test correction for multiple testing)

We then measured cilia length and incidence in RP11 - RPE cells using a combination of ARL13B and a basal body marker (pericentrin) that is located at the base of the cilia (Fig. 5a). Both cilia incidence and cilia length were significantly reduced in all RP11 - RPE cells when compared to controls (Fig. 5b). TEM analysis revealed the presence of long cilia with clearly aligned microtubules in control RPE cells, while RP11 - RPE cells had shorter, abnormal, bulbous cilia (Fig. 5c). Structural defects in axonemal microtubules were confirmed by serial block face scanning electron microscopy (SBFSEM, Fig. 5d). RP11 photoreceptors also had significantly reduced cilia incidence (Fig. 5e, f) and defective, bulbous cilia with misaligned microtubules (Fig. 5g) that was also confirmed by SBFSEM analysis (Fig. 5h).

**Fig. 6:**
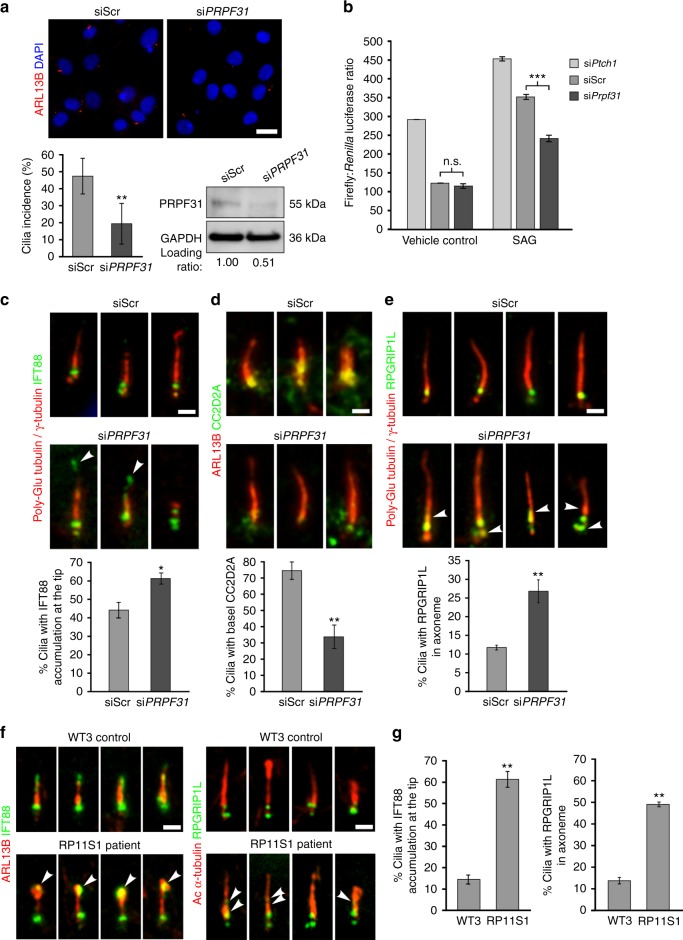
PRPF31 loss causes defects in cilia incidence and structural organisation. **a** *PRPF31* siRNA knockdown in human hTERT-RPE1 cells causes a significant decrease in cilia incidence (lower left) and PRPF31 protein levels (lower right) compared to scrambled negative control (siScr) siRNA, scale bar: 10 μm; **b** Gli1 reporter assays of Shh activity measured in NIH3T3-GL cells following knockdown for *Ptch1* (positive control), scrambled negative control siRNA (siScr) and *Prpf31*. Cells were treated with either 100 nM SAG or vehicle control for 48 h, as indicated. Assays results are expressed in arbitrary units of the ratio of firefly: *Renilla* luciferase activities; **c** Ciliary localisation of IFT88 (green) in primary cilia of hTERT-RPE1 cells (visualised by staining for γ-tubulin and poly-glutamylated tubulin; red) showing mislocalisation of IFT88 (arrowheads) at ciliary tips following *PRPF31* knockdown. Bar graph quantitates the percentage of cilia with IFT88 at their tip. Scale bar: 1 μm; **d** Visualisation and quantitative analysis of the transition zone protein CC2D2A (green) and ARL13B (red); **e** Visualisation and quantitative analysis of the transition zone (TZ) protein RPGRIP1L (green) and cilia (γ-tubulin and poly-glutamylated tubulin; red) showing mislocalisation of RPGRIP1L from the TZ into the ciliary axoneme (arrowheads) following *PRPF31* knockdown. **f**, **g** Ciliary localisation of IFT88 and RPGRIP1L (green) in RP11 - RPE cells showing mislocalisation of IFT88 (arrowheads) at ciliary tips and RPGRIP1L from the TZ into the ciliary axoneme (arrowheads). **a**–**g** Data shown as mean ± SEM, *n* = 3. Statistical significance of pairwise comparisons is indicated by n.s. not significant; *p < 0.05; **p < 0.01; ***p < 0.001 (Student’s unpaired *t* test). **c**–**f** Scale bar: 1 μm

To further confirm that loss of human PRPF31 negatively regulates ciliogenesis, we performed siRNA knockdown in the human ciliated retinal pigment epithelial hTERT-RPE1 cell line. Knockdown of PRPF31 protein levels caused a significant decrease in cilia incidence (Fig. 6a). Since SHH activity is known to require functional cilia, we confirmed that *PRPF31* siRNA knockdown caused a dysregulated response to Smoothened agonist (SAG; Fig. 6b). To investigate possible defects in ciliary morphogenesis and structural organisation as a consequence of ciliary gene mis-splicing, we used structured illumination microscopy (SIM) to resolve the detailed localisation of proteins along the ciliary axoneme and at the transition zone (TZ). *PRPF31* knockdown caused significant mislocalisation of IFT88 to the ciliary tip (Fig. 6c), and the TZ proteins CC2D2A and RPGRIP1L were either entirely excluded from the TZ (Fig. 6d) or mislocalised from the TZ into the ciliary axoneme (Fig. 6e). Similar mislocalisation was also evident in RP11 - RPE (Fig. 6f, g).

### Correction of *PRPF31* mutation restores molecular and cellular defects

**Fig. 7:**
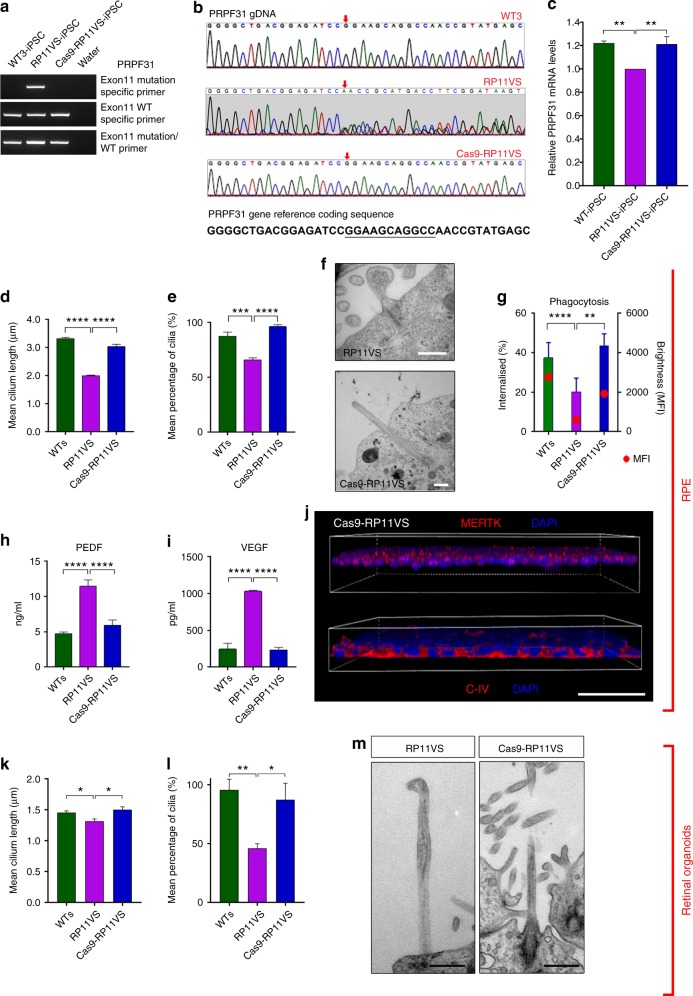
Gene correction of *PRPF31* mutation results in reversal of cellular and functional phenotypes in RPE and photoreceptors. **a**–**c** CRISPR/Cas9 correction of the *PRPF31* deletion in exon 11; **d**, **e** Quantification of cilia length and incidence in *PRPF31-* and WT-RPE. **f** TEM analysis of *PRPF31*-edited RPE cilia showing morphologically normal cilia, scale bar: 500 nm; **g** Increased phagocytosis in *PRPF31*-edited RPE. **h**–**j** Restoration of apical–basal polarity in *PRPF31*-edited RPE, scale bar: 50 μm. **k**, **l** Quantification of cilia length and frequency in *PRPF31*- and WT-photoreceptors; **m** TEM analysis of *PRPF31*-edited photoreceptor cilia showing morphologically normal cilia, scale bar: 500 nm. **c**–**e**, **g**–**i**, **k**, **l** Data shown as mean ± SEM, *n* = 3. Statistical significance of pairwise comparisons is indicated by n.s.: not significant; *p < 0.05; **p < 0.01; ***p < 0.001; ****p < 0.0001 (Student’s paired *t* test)

To further validate the function of PRPF31 in retinal cells, CRISPR/Cas9 genome editing was used to correct the *PRPF31* c.1115_1125del11 genetic mutation in cells from the patient with the most severe clinical phenotype (RP11VS). For in situ gene correction, an ssODN template with wild-type *PRPF31* sequences was designed with 91 bp homology arms on each side of the mutation region (Supplementary Data 6). Two hundred iPSC clones were selected and tested (Fig. 7a), and candidates identified by PCR were sequenced to confirm gene editing of *PRPF31* (Fig. 7b). Quantitative RT-PCR analysis confirmed the increased expression of *PRPF31* in the CRISPR/Cas9-corrected clone when compared to uncorrected iPSCs (Fig. 7c). We also excluded potential off-target effects (Supplementary Data 7) and CytoSNP analysis confirmed the identity to the parental cell line and lack of genomic abnormalities (Supplementary Figure 8). The CRISPR/Cas9 iPSC clone expressed pluripotency-associated markers Nanog and TRA-1-60, and gave rise to cells belonging to all three germ layers (Supplementary Figure 9).

The CRISPR/Cas9-corrected iPSC clone was differentiated to RPE and retinal organoids, in parallel with uncorrected RP11-iPSCs using our established protocols. Cilia length and incidence was significantly increased in both corrected RPE (Fig. 7d, e) and corrected photoreceptors (Fig. 7k, l). TEM analysis also revealed cilia with well-aligned axonemal microtubules in corrected cells that did not display the aberrant morphology observed in the RP11-derived retinal cells (Fig. 7f, m). Importantly, flow cytometry confirmed the rescue of phagocytic capacity (Fig. 7g), suggesting an improvement of functional characteristics in corrected RPE. Immunostaining and cytokine secretion assays also revealed the restoration of cytokine secretion and basal collagen IV and apical MERTK expression, suggesting that corrected RPE apical – basal polarity was restored (Fig. 7h–j). These data indicate that in situ gene editing restored key cellular and functional phenotypes associated with RP type 11.

To further assess the impact of alternative splicing on protein abundance, quantitative proteomic analysis was carried out in RP11VS and CRISPR/Cas9-corrected RPE and retinal organoids using TMT labelling and mass spectrometry (Supplementary Figure 10A-F and Supplementary Data 8, 9). GO enrichment analysis for biological processes indicated that RNA metabolic processes, mRNA processing, RNA splicing, and both RNA and DNA metabolic processes, to be the most affected pathways in RPE and retinal organoids, respectively (Supplementary Figure 10A, D). Several components of the mRNA surveillance pathway (MSI2 and RNPS1), the PRP19 complex (PLRG1 and CTNNBL1), the SF3a/SF3b complex (SF3A1 and SF3B4), spliceosomal tri-snRNP proteins (PRPF3, USP39, PRPF6 and DDX23) and SR proteins (SRSF1, 2, 5 and 6) were downregulated in either RP11 patient-specific RPE, retinal organoid cells or both (Supplementary Data 8, 9). The endoplasmic reticulum, nucleoplasm, ribonucleoprotein and spliceosomal complex were the most affected cellular components (Supplementary Figure 10B, D), corroborating the splicing deficiency highlighted by our RNA-Seq and splicing assays. Ten per cent of the differentially expressed genes and 10% of differentially spliced transcripts showed differential protein expression in RPE cells (Supplementary Figure 10F). Of the 49 differentially spliced transcripts and differentially expressed proteins, nine were associated with the ribonucleoprotein complexes and shown to be involved in pre-mRNA splicing, RNA binding and translation initiation, further corroborating the impact of *PRPF31* mutations on the spliceosome complex. The impact of differential gene expression and exon usage was less pronounced in retinal organoids than in RPE cells (1.6% and 0.74%, respectively) (Supplementary Figure 10C). Of the 14 differentially spliced and expressed proteins, PRPF31 itself was identified, in addition to superoxidase dismutase mitochondrial protein (SOD2) for which reduced expression has been linked to retinopathies. The latter was also significantly downregulated in mutant RPE cells. Collectively, the proteomic data suggest that differential splicing may play a more significant role in protein isoform generation in RPE when compared to retinal organoids. This data highlights key candidate genes and proteins that are affected by alternative splicing in RP11 retinal cells and deserve further investigation.

## Discussion

Retinitis pigmentosa (RP) is one of the most common forms of hereditary progressive sight loss. Autosomal-dominant inheritance accounts for about 40% of RP, with an estimated 15% of cases of this RP inheritance type caused by mutations in pre-mRNA processing factors (PRPFs). PRPFs are ubiquitously expressed and involved in the formation of stable U4/U6.U5 tri-snRNPs and the spliceosomal B complex leading to spliceosome activation, yet human *PRPF* mutations result in retinal-specific phenotypes. Despite a large body of work in immortalised cell lines and animal models, there are no described cellular phenotypes for *PRPF31*-related RP type 11 that define the primary affected cell type or provide clear insights into the pathomechanisms that can explain the retinal specificity of phenotypes.

**Fig. 8:**
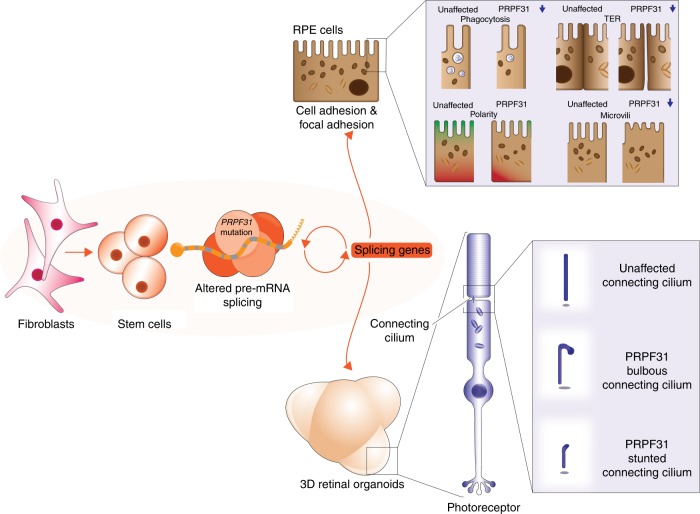
Schematic diagram that summarises pathogenic pathways in the RP11 disease state. Altered pre-mRNA splicing in iPSC-derived RPE and retinal organoids is associated with severe RPE defects and disrupted cilia in photoreceptors

To gain insights into RP pathomechanisms, we characterised the cellular phenotypes and splicing programmes of RPE and retinal organoids in comparison to fibroblasts and iPSCs derived from RP11 patients with *PRPF31* mutations. Through large-scale transcriptome and biochemical analyses, we provide evidence that impaired in vivo splicing is restricted to patient-derived retinal cells only, and that impaired pre-mRNA splicing appears to be limited to splicing programmes that affect RNA processing itself (Fig. 4). These splicing defects appeared to be correlated with ultrastructural, cellular and functional deficiencies that are characteristic of RPE in the RP disease state. These include shorter microvilli and primary cilia, loss of polarity, reduced barrier function and defective phagocytic capacity. Similarly, photoreceptors in patient-specific retinal organoids had defective primary cilium morphology and features of degeneration and cell stress. This was corroborated by transcriptome analyses of *Prpf31*^+/−^ mouse tissues, demonstrating that alternatively spliced transcripts in retinae and RPE (but not brain or muscle) also occurred within the pre-mRNA splicing category (Fig. 4e, f). Our results are consistent with the existence of precisely regulated mRNA splicing programmes in the normal mouse retina that are essential for the formation of primary cilia and their subsequent development into photoreceptor outer segments^12^. The exacerbation of splicing deficiencies in RP11 retinal cells, in addition to the disruption of splicing programmes for ciliary genes, are likely to cause the serious deleterious effects on cellular phenotypes that underlie splicing factor RP clinical phenotypes. Importantly, our transcriptomics and proteomics analysis suggest that RPE is the most severely affected retinal cell type in *PRPF31*-mutated RP11 patients. In our previous work, we have shown that reduced cilia length in fibroblasts derived from RP11 patients;^34^ however, studies to date have not highlighted cellular deficiencies outside the retina. Despite this ciliary deficiency, we were able to reprogramme RP11 fibroblasts to iPSC with similar efficiency and ease as the unaffected controls. Thus, we suggest that altered pre-mRNA splicing is the primary pathogenic defect and cilia deficiencies are secondary impacts that arise from splicing deficiencies (Fig. 8). Since a recent manuscript has shown that *PRPF8* defects cause mis-splicing in myeloid malignancies^35^, it will be important in future studies to investigate cellular phenotypes in tissues that have not previously been noted to have clinical manifestations of pre-mRNA splicing factor deficiency.

Our transcriptome analyses do not directly interrogate the consequences of mis-splicing at the protein-coding level. However, our quantitation of the different types of alternative splicing events in cell types (Fig. 4a) showed that RPE and retinal organoids had the highest burden of retained intron (RI) events. Our transcriptome analyses do not interrogate the levels of non-canonical or intergenic transcripts that could arise from retinal cell splicing deficiencies, since the analysis software only aligns and maps sequence reads to annotated alternative transcripts. Unique unmapped reads comprised a minority of total reads (mean = 11.307%, SD = 3.569%, range 5.917–20.797%), but there is unlikely to be a potential contribution of non-canonical transcripts to RP cellular phenotypes because there are consistently no significant differences in the proportion of unmapped reads between control and disease data sets (*p* = 0.57, unpaired *t* test). However, our use of polyA-selected RNA rather than total RNA could not exclude the involvement of other classes of noncoding or non-canonical transcripts such as microRNAs, long noncoding RNAs and circular RNAs. Recent work has shown that the output of protein-coding genes shifts to circular RNAs by a process of back-splicing under conditions when pre-mRNA processing components are limiting^36^, but the relative contribution of this process to RP pathogenesis cannot be determined from our data sets. However, our in vitro and in vivo data sets both strongly suggest that the exacerbation of splicing deficiencies, specifically of canonical transcripts in retinal cells, contributes to the restricted retinal phenotype in RP11 patients.

Our study recruited four patients with different clinical severity and although this is a small number for performing phenotype–genotype correlations, we observed some differences in cellular phenotypes. For example, there was no significant reduction in phagocytosis for RPE cells derived from the patient with mild clinical severity. The incomplete penetrance of RP11 has been linked to the number of microsatellite repeat elements (MSR1) adjacent to the *PRPF31* core promoter^37^, but we did not find any difference in the number of MSR1 repeats between the patients and controls. Variable clinical severity has also been linked to the expression of CNOT3, which acts as genetic modifier for *PRPF31*^38^. The inclusion of only four patients makes it difficult to assess such genetic correlations in the present study, but our work provides guidelines about the optimal size and direction of larger future investigations into correlations between cellular phenotype and genotype.

The development of therapies for RP and other retinopathies is a key goal in the stem cell and regenerative medicine fields. CRISPR/Cas9-mediated in situ gene editing has become a popular methodology for correcting mutations in iPSCs prior to differentiation into cells of interest. Our iPSC-derived disease models are the first for this type for splicing factor RP, and we were able to correct the mutation in iPSC cells derived from a RP11 patient with the most severe clinical phenotype (Fig. 7a–m). This rescued all key cellular and functional phenotypes in RPE, the most severely affected patient cell type, without causing off-target effects. This demonstrates proof of concept that in situ gene editing is effective. For this approach to be effective in RP patients, in situ gene editing in patient′s RPE and photoreceptors has to be performed. This strategy has been successfully tested in animal models of RP^39^ and intense efforts are under way to make this applicable for human clinical trials^40^. In addition to CRISPR/Cas9 gene editing, restoration of *PRPF31* mRNA and protein expression could also be achieved by traditional gene therapy approaches using AAV vectors that have been optimised for retinal cells^41^. Notwithstanding, our studies showed that *PRPF31*-mutated RPE had low but detectable levels of mutant PRPF31 proteins, corresponding to the long and NMD-insensitive mutant (Fig. 3c). It is unclear if mutant PRPF31 proteins could have a dominant-negative effect, either compromising spliceosome function in a cell-type-specific fashion, or causing differences in cellular phenotypes between iPSC-derived lines and even retinal phenotypes in patients. In contrast, PRPF31 mutant proteins were not detected in patient-specific retinal organoids, which suggests that any cellular and functional impairment in photoreceptors can be ascribed to PRPF31 haploinsufficiency, which is consistent with previous studies^42^. Nevertheless, the presence of PRPF31 mutant proteins in RPE suggests that the use of allele-specific antisense or morpholino oligonucleotides will be required as an additional treatment strategy to modify *PRPF31* gene expression in order to more fully rescue all retinal phenotypes in splicing factor RP. However, there are important caveats to extrapolating disease modelling in iPSCs and derivatives to future preclinical studies of RP. In particular, cellular phenotypes of RP are detectable in the iPSC-derived retinal cell types weeks after differentiation, whereas the clinical phenotype in RP patients manifests as a late-onset condition. Part of this disparity may be explained by the observation of rapid premature senescence in RPE differentiated from iPSCs^43,44^.

In conclusion, our data provide a detailed mechanistic explanation of retinal-specific phenotypes in *PRPF31*-mutated RP type 11 (summarised in Fig. 8) and, more generally, the characterisation of potential pathomechanisms during retinal degeneration. Our transcriptome data sets comprise a comprehensive catalogue of target genes affected by *PRPF31* mutations. These delineate retinal-specific splicing programmes in the RP disease state, providing new insights into the contribution of mRNA processing to human disease.

## Methods

### Human subjects

All samples used in this study were obtained with informed consent according to the protocols approved by Yorkshire and the Humber Research Ethics Committee (REC ref. no. 03/362). Further information on the patients and controls is provided in Methods and in Supplementary Data 1.

### Animals

The in vivo experiments using mice were performed according to protocols approved by the Institutional Animal Care and Use Committee of the Massachusetts Eye and Ear Infirmary. All procedures were performed to minimise suffering in accordance with the animal care rules in the institution in compliance with the Animal Welfare Act, the Guide for the Care and Use of Laboratory Animals, and the Public Health Service Policy on Humane Care and Use of Laboratory Animals.

### iPSC generation

Three age-matched unaffected controls (WT1, WT2 and WT3) and four RP11 dermal skin fibroblasts (RP11M, RP11S1, RP11S2 and RP11VS, Supplementary Data 1) were cultured with advanced Dulbecco’s modified Eagle Medium (Thermo-Fisher, Waltham, MA, USA) containing 10% FBS (Thermo Fisher Scientific), 1% glutamax (Thermo Fisher Scientific) and 1% penicillin/streptomycin (Thermo Fisher Scientific) at 37 °C and 5% CO_2_ in a humidified incubator. These fibroblasts were transduced at a density of 30,000 cells/cm^2^ using the CytoTune™-iPS 2.0 Reprogramming Kit (Life Technologies, A16517) following the manufacturer’s instructions. iPSC colonies were established on inactivated primary mouse embryonic fibroblasts feeder layer and then adapted to the feeder-free system described below.

### iPSC culture

Human iPSCs were cultured on six-well plates on Matrigel™ GFR (Corning, 354230)-coated wells with mTeSR™1 (StemCell Technologies, 05850) media supplemented with penicillin/streptomycin (Gibco, 15140). Cell culture medium was replaced on a daily basis. Cells were allowed to grow for 4–5 days prior to passaging or induction of differentiation. Passaging was carried out using Versene (EDTA 0.02%) (Lonza, BE17–771E) solution at 37 °C for 3–5 min and cells were transferred to fresh matrigel plates in a 1:3–1:6 ratio. All cultures were maintained at 37 °C, in a humidified environment, with 5% CO_2_. Cells were cryopreserved with freezing media containing 90% foetal bovine serum (Gibco, 10270) and 10% dimethyl sulfoxide (Sigma, D2650).

### Detection of pluripotency markers by immunocytochemistry

iPSC colonies were fixed in 4% paraformaldehyde (Sigma, 47608) for 15 min at room temperature and permeabilised with 0.25% Triton X-100 (Sigma, T8787) for 40 min. Blocking solution was applied (10% FBS + 1% bovine serum albumin—Sigma, A3311) for 45 min at room temperature before proceeding with addition of anti-human SSEA4 conjugated with Alexa Fluor® 555 (BD Biosciences, 560218, 1:200) and anti-human OCT4 primary antibody (R&D, AF1759, 1:200). Secondary staining was performed with the antibody anti-goat IgG with FITC (Jackson Immuno Research, 705-096-147, 1:500) diluted in blocking solution, followed by nuclear counterstaining with DAPI (Partec, 05-5005). Colonies were imaged using a Bioscience Axiovert microscope in combination with the associated Carl Zeiss software, AxioVision. All antibody details are shown in Supplementary Data 6.

### Detection of pluripotency markers by flow cytometry

iPSCs were treated with 0.02% EDTA (Lonza, BE17-711E) for 3 min at 37 °C to dissociate the colonies. The suspension was collected in phosphate buffer saline (PBS) and centrifuged for 3 min at 300 × *g*. Supernatant was removed and replaced with PBS with 0.1% BSA containing TRA-1-60-conjugated FITC (Merck Millipore, FCMAB115F, 1:60) and NANOG conjugated with Alexa Fluor® 647 (Cell Signaling Technology, 5448S, 1:150). Samples were incubated in the dark at room temperature for 60 min on a shaker. Cells were washed with PBS and resuspended in FACS buffer (PBS with 2% FBS). At least 10,000 events were analysed using a FACS Canto II flow cytometer. Results were analysed using the FACSDiva software.

### In vitro and in vivo three germ-layer differentiation

iPSCs were detached from six-well plates (20–30 colonies per well) using 1 ml of 1 µg/ml Collagenase type IV (Gibco 17104–019) and 0.5 µg/ml Dispase II (Gibco, 17105–041) solutions. The colony suspension was transferred to a 50 ml conical tube until the colonies settled in the bottom of the tube. The supernatant was carefully aspirated and 2 ml of differentiation media, containing DMEM-F12 (Gibco 11330), 20% FBS (Gibco, 10270), 1% penicillin/streptomycin (Gibco, 15140), 1% non-essential amino acids (Gibco, 11140), was added per well. The colony suspension was then transferred to a 10 cm Petri dish and media was changed every day. After 7 days, the embryoid bodies (EBs) were transferred to a gelatin-coated 24-well plate or a chamber slide. After an additional 7 days, colonies were fixed and stained with specific antibodies for the three germ layers using the 3-Germ Layer Immunocytochemistry Kit (Life Technologies, A25538). As a negative control, cells were stained only with secondary antibodies.

For the teratoma assay, iPSC colonies were dissociated with EDTA and 1 million cells were resuspended in a 200 μl solution of PBS (Gibco, 14190) + 2% FBS (Gibco, 10270). The samples were injected intraperitoneally in immunosuppressed mice at the Comparative Biology Centre, at the Medical School, Newcastle University. Each injection consisted of 0.5 million cells mixed with 100 μl Matrigel (BD, 354230). Following a period of 10 weeks, the mice were killed and the teratomae were excised, processed and sectioned according to standard procedures and stained for Weigert’s haematoxylin, Masson’s trichrome and Mayer’s haematoxylin and eosin histological analysis. Sections (5–8 µm) were examined using bright-field microscopy and stained tissue photographed as appropriate.

### hTERT-RPE1 cell culture

Human telomerase reverse transcriptase-transformed retinal pigment epithelium (hTERT-RPE1) were purchased from American Type Culture Collection (ATCC) at passage 9. The genomic status of the cell lines was assessed by array CGH and karyotyping. All cell lines were tested every 3 months for mycoplasma. Cell lines were maintained in DMEM/Ham’s F12 medium (Gibco, 31331) supplemented with 10% foetal calf serum (FCS) (Sigma-Aldrich, F7524), under standard conditions (37 °C, 5% CO_2_). Cells were passaged at a split ratio of 1:8 twice a week, with low passages (<25) for both mIMCD3 and hTERT-RPE1 cells. hTERT-RPE1 cells were serum starved in OptiMEM (Gibco, 31985) for 48 h to induce ciliogenesis.

### RNA isolation and reverse transcription

iPSC and iPSC-derived cell pellets were washed with PBS before being lysed with RNA Lysis buffer provided by the RNA extraction kit ReliaPrep^TM^ RNA Cell Miniprep System (Promega, Z6010). The manufacturer’s instructions were followed, including a DNAse incubation step to the extracted RNA. The products were then passed through a column and resuspended in nuclease-free water. RNA was stored at −80 °C or immediately used for cDNA synthesis. RNA was measured with a NanoDrop 2000 Spectrophotometer (Thermo Scientific) and 1 µg of extracted RNA was converted into cDNA using GoScriptTM Reverse Transcription System (Promega, A5000) following the manufacturer’s instructions.

### Reverse transcription polymerase chain reaction

For detection of any residual expression of the ectopically applied Yamanaka factors, RT-PCR was utilised in which the primers used were complementary to part of the SeV vector as well as the transgenes. Oligonucleotides for the housekeeping gene (*GAPDH*) were used as a positive control. For the detection of mRNA transcripts as a result of c.1115_1125 del11 mutation, we have designed primers to detect wild type, long and short mutant transcripts of *PRPF31* gene. All primers are listed in Supplementary Data 6. For the PCR reaction mixture, cDNA produced from 1 µg of RNA was amplified using the primers described on CytoTune™-iPS 2.0 Sendai Reprogramming Kit User Guide at the concentration of 10 µM each in addition to 10 µM dNTP mix, 5X Green GoTaq® Reaction Buffer and GoTaq® DNA Polymerase (5 U/µl) (Promega, M3175). The PCR consisted of a 35-cycle programme of 95 °C for 30 s, 55 °C for 30 s followed by 72 °C for 30 s and was carried out using a Mastercycler® thermal cycler. Following the reaction, the samples were analysed using a 2% agarose gel electrophoresis mixed with GelRedTM Nucleic Acid Stain (Biotium, 41003). A 100 bp ladder was run against the samples.

### Quantitative real-time polymerase chain reaction

qRT-PCR was performed using the GoTaq™ qPCR Master (Promega) according to the manufacturer’s instructions. Each reaction contained 5 μl GoTaq qPCR Master Mix (Promega), 0.5 μl cDNA sample, nuclease-free water and 0.6 μl primers (10 μM). All amplified products ranged from 100 to 200 bp in size. The plates were run on an Applied Biosystems 7500 fast Real Time PCR machine. The cycling programme consisted of a hot-start activation at 95 °C for 5 min, followed by 45 cycles of denaturation at 95 °C for 10 s, annealing/extension at 60 °C for 30 s and denaturation 95 °C for 1 min. Following amplification, a melt-curve analysis was performed from 65 to 95 °C with 0.5 °C increments every 10 s. Each sample was run in triplicate, and the average quantification cycle (Cq) value was determined. Control reactions were run with water instead of template for each primer pair to check for primer-dimers and reagent contamination. Normalised gene expression values (against *GAPDH*) were obtained using the ΔΔCT method. All primer details are shown in Supplementary Data 6.

### Genomic DNA extraction

Genomic DNA was extracted from the pelleted cultures of the iPSC and corresponding parental fibroblast cell lines using the QIAamp DNA Mini Kit (Qiagen, 56304) following the manufacturer’s instructions.

### Mutation screening

An aliquot of 10 ng of DNA from control and patients’ fibroblasts and iPSCs was amplified by standard PCR (40 cycles of 95 °C for 30 s, 64 °C for 30 s and 72 °C for 30 s) using primers described by Dong et al.^42^ for the specific exons where the *PRPF31* mutations were located. The amplified products were purified using the QIAquick PCR Purification Kit (Qiagen, 28104) and quantified using the Qubit® 2.0 Fluorometer. The sequencing files were analysed in the SeqScape v.2.5 software and forward and reverse sequences from both fibroblasts and iPSCs were aligned and compared with the *PRPF31* reference sequence gene (NG_009759.1) from GenBank to identify the *PRPF31* mutations. The consensus sequences from the forward and reverse sequences were then extracted from the software and pairwise aligned against the coding *PRPF31* sequence. Here the nucleotide designated as 1 commences at position 36 of GenBank accession number AL050369. All primer details are shown in Supplementary Data 6.

### SNP array

DNA samples from the iPSCs and corresponding parental fibroblasts were analysed using the Infinium HumanCytoSNP-12 (Illumina, WG-320-2101) SNP array following the manufacturer’s instructions. The results were analysed using the BlueFuse Multi 4.3 software (Illumina, San Diego, USA).

### iPSC differentiation to retinal pigment epithelium

iPSC colonies were grown to 80–95% confluency and all differentiation areas were removed. mTeSR™1 media was replaced with 2 ml of differentiation medium [Advanced RPMI 1640, (12633, Gibco), GlutaMAX-1 (35050, Gibco), penicillin/streptomycin (Gibco, 15140) and B-27 (Gibco, 17504)] supplemented with 10 μM SB431542 (STEMCELL™, 72232) and 10 ng/μl Noggin (R&D Systems, 6057-NG-025) from days 0 to 5. From days 6 to 9, only 10 ng/μl Noggin (R&D Systems, 6057-NG-025) was added to the medium. From days 10 to 15, the medium was supplemented with 5 ng/μl Activin A (PeproTech, 120–14 A) and from days 16 to 21, Activin A was replaced with 3 μM CHIR99021 (Sigma, SML1046). The cells were then fed every 2 days until the first RPE patches appeared, normally by week 4 of differentiation. RPE patches were mechanically picked and placed in TryPLE (10×) (Invitrogen, USA) for 30 min to dissociate the cells, agitated by gentle pipetting at 10, 20 and 30 min. Cells were sieved using a 100 µm cell strainer and re-plated at 4.5 × 10^5^ cells per cm^2^ on 24-well plates or 0.33 cm^2^ PET hanging cell culture inserts (Merck Millipore; Billerica, USA) coated with PLO/laminin (50 ng/μl) (Sigma-Aldrich, USA).

### iPSC differentiation to retinal organoids

The method for generating retinal organoids from iPSC was based on a previously described protocol^24^ with minor modifications. Briefly, iPSCs were dissociated into single cells using Accutase (Gibco, A1110501). iPSCs were re-aggregated using low-cell adhesion 96-well plates with U-bottomed conical well (Lipidure® COAT Plates, NOF Corp.) at a density of 12,000 cells/well in mTeSR1 media supplemented with ROCK inhibitor (Y-27632, Chemdea, CD0141, 20 µM). After 48 h, the media was changed to differentiation medium (45% Iscove’s modified Dulbecco’s medium (Gibco, 12440–053), 45% Hams F12 (Gibco, 31765–029), 10% KSR (Gibco, 10828–028), glutamax (Gibco, 35050–038), 1% chemically defined lipid concentrate (Thermo, 11905031), 450 µM monothioglycerol (Sigma, M6145), penicillin/streptomycin (Gibco, 15140–122). This was defined as day 0 of differentiation. BMP4 (55 ng/ml, R&D, 314-BP) was added to the differentiation medium on day 6. Half of the medium was exchanged every third day. On day 18, the aggregates were transferred from a 96-well plate to a low-attachment 6-well plate, and further cultured in suspension in the neural retinal differentiation medium containing DMEM/F12 (Gibco, 31330–038), 10% foetal bovine serum (Gibco,10270–106), 1% N2 supplement (Thermo, A1370701), 0.1 mM taurine (Sigma, T8691), 0.5 µM retinoic acid (Sigma, R2625), 0.25 μg/ml Fungizone (Gibco, 15290–02), penicillin/streptomycin (Gibco, 15140–122). The cells were maintained for up to 43 weeks, with media changes every 3–4 days.

### APS-MEA experiments

Recordings were performed on the BioCam4096 platform with BioChips 4096S+ (3Brain GmbH, Lanquart, Switzerland), integrating 4096 square microelectrodes. Organoids were transferred to 33 °C artificial cerebrospinal fluid (aCSF) containing the following (in mM): 118 NaCl, 25 NaHCO_3_, 1 NaH_2_ PO_4_, 3 KCl, 1 MgCl_2_, 2 CaCl_2_, 10 glucose, and 0.5 l-glutamine, equilibrated with 95% O_2_ and 5% CO_2_. Organoids were dissected longitudinally and placed, with the presumed RGC layer facing down, onto the 4096 channel MEA, flattened with a translucent polyester membrane filter (Sterlitech Corp., Kent, WA, USA). The organoids were allowed to settle for at least 2 h. To reliably extract spikes from the raw traces, we used a quantile-based event detection^45^ and single-unit spikes were sorted using an automated spike sorting method for dense, large-scale recordings^46^. Statistical significance and firing rate analyses were evaluated by using Prism (GraphPad, CA) and MATLAB (Mathworks, MA). Light stimuli were projected as described previously^46^. Broad white light pulses (200 ms, 217 µW/cm^2^ irradiance, 1 Hz) were presented for 5 min onto the organoids after recording of 5 min without pulsed light stimulation. The drugs cGMP (8-Bromoguanosine 3′,5′-cyclic monophosphate, Sigma-Aldrich, MO) and GABA (γ-Aminobutyric acid, Tocris Bioscience, Bristol, UK) were puffed in the recording chamber (final concentrations, cGMP 100 µM, GABA 125 µM) and 2 min before and after the puff were recorded.

### CRISPR-Cas9 correction of *PRPF31* mutation in the RP11VS

Correction of *PRPF31* mutation in the RP11VS iPSCs was achieved by using the CRISPR/Cas9 system in combination with ssODNs as homologous templates covering the mutation site. The online design tool (http://tools.genome-engineering.org) was used to design the sgRNA sequences and predict off-targets. The sgRNA (see Supplementary Data 6), which targets only mutant but not wild-type *PRPF31* sequences and predicted to have low off-targets, was chosen. The sgRNA was cloned into the CRISPR/Cas9 vector (pSpCas9(BB)-2A-Puro) following the protocol from Ran et al.^47^. The ssODN template with wild-type *PRPF31* sequences was designed manually with 91 bp homology arms on each side of the mutation region (Supplementary Data 6). The sgRNA-CRISPR/Cas9 vector and ssODN were co-transfected into the RP11VS iPSCs by using Lipofectamine-3000 (Invitrogen) according to the manufacturer’s instructions. Twenty-four hours after transfection, puromycin (0.2 µg/ml) was added for 2 days. Four–5 days after selection, the resistant iPSCs were dissociated into single cells using Accutase (Gibco, A1110501). A total of 100,000 cells in mTeSR1 media supplemented with ROCK inhibitor (Y-27632, Chemdea, CD0141, 20 µM) were plated on a 10 cm Matrigel-coated dish. After 7 days, the colonies were picked and transferred to a cell culture 96-well plate. When the wells became confluent, iPSCs were split in two 24-well plates for further expansion and DNA isolation. Genomic DNA (gDNA) was isolated using QIAamp DNA Mini Kit (Qiagen, 56304). Subsequently, PCR were performed with the primers including *PRPF31*-mutation specific primers; *PRPF31*-WT-specific primers; *PRPF31*-mutation/WT primers (Supplementary Data 6). The positive clones, which are negative for *PRPF31*-mutation-specific primers and positive for *PRPF31*-WT-specific primers were sequenced to confirm in situ gene editing of *PRPF31*.

### Off-target prediction and capture sequencing

sgRNA off-target sequences were predicted using the online design tool (http://tools.genome-engineering.org). Each sgRNA off-target sequence was blasted against the human genome reference (https://blast.ncbi.nlm.nih.gov/Blast.cgi). Capture intervals were expanded by ~500 bp in both the 5′ and 3′ directions. Primers were designed in this region (Supplementary Data 6). The PCR products were then sequenced to check the off-target effects of sgRNA.

### Measurement of trans-epithelial resistance

TER was performed using a Millicell ERS-2 Voltohmmeter (Millipore, MERS00002) by measuring the resistance of the blank transwell insert with PBS (Gibco, 14190) and the insert with RPE cells. The shorter and longer tips of the electrode were inserted in the transwell apical chamber and in the basolateral chamber, respectively. The resistance was measured twice in each transwell insert. The resistance reading of the blank was then subtracted from the resistance reading of the cells for each measurement. The results were multiplied by the membrane area value using the formula: Unit area resistance = Resistance (Ω) × effective membrane area (cm^2^), where the final value was given in ohms (Ω).

### Transmission electron microscopy

RPE and 3D optic cup samples were fixed with 2% gluteraldehyde in 0.1 M sodium cacodylate buffer and sent to the transmission electron microscopy facilities at Newcastle University, where samples were post fixed in 1% osmium tetroxide, dehydrated in gradient acetone and embedded in epoxy resin. Ultrathin sections (70 nm) were picked up on copper grids, stained with uranyl acetate and lead citrate and imaged using a Philips CM100 transmission electron microscope with high-resolution digital image capture.

### Serial block face SEM

Cells were fixed overnight in 2% glutaraldehyde in 0.1 M sodium cacodylate buffer. Once fixed, the samples were processed using the heavy metal staining protocol adapted from Deerinck et al.^48^. Briefly, samples were incubated in a series of heavy metal solutions −3% potassium ferrocyanide in 2% osmium tetroxide, 10% thiocarbohydrazide, 2% osmium tetroxide again, 1% uranyl acetate overnight and finally lead aspartate solution. Between each step the samples were rinsed thoroughly in several changes of deionised water. Samples were dehydrated through a graded series of acetone and then impregnated with increasing concentrations of Taab 812 hard resin, with several changes of 100% resin. The samples were embedded in 100% resin and left to polymerise at 60 °C for a minimum of 36 h. The resin blocks were trimmed to ~0.75 mm by 0.5 mm and glued onto an aluminium pin. In order to reduce sample charging within the SEM, the block was painted with silver glue and sputter coated with a 5 nm layer of gold. The pin was placed into a Zeiss Sigma SEM incorporating the Gatan 3view system, which allows sectioning of the block in situ and the collection of a series of images in the *z*-direction. Multiple regions of interest were imaged at ×2000 magnification, 3000 × 1500 pixel scan, which gave a pixel resolution of ~15 nm. Section thickness was 50 nm in the *z*-direction. In the resulting z-stacks, cilia were identified and segmented manually using Microscopy Image Browser (MIB, University of Helsinki). The segmentations were imported into Amira (FEI) for construction of the 3D models.

### Phagocytosis assay

Bovine rod photoreceptor outer segments (POS) (InVisionBioResources, 98740) were centrifuged at 2600 × *g* for 4 min and the pellet was resuspended in 100 μl of advanced RPMI (AdRPMI) 1640 medium (12633, Gibco). The POS were incubated with 0.4 mg/ml FITC (Sigma, F7250) for 1 h at room temperature and agitated in the dark. POS were centrifuged at 2600 × *g* for 4 min and washed three times with PBS (Gibco, 14190). Then, they were resuspended in AdRPMI 1640 (12633, Gibco) + B-27 Supplement (Gibco, 17504) + 10% foetal bovine serum (FBS) (Gibco, 10270) and the staining was confirmed under a Bioscience Axiovert microscope. RPE cells were treated with 1 × 10^6^ POS-FITC per cm^2^ and incubated for 4 h at 37 °C. For the control experiments, RPE cells were treated with the same number of non-stained POS and incubated for the same time. Cells were rinsed with PBS supplemented with calcium and were detached from the wells using 200 μl of Trypsin for 5–8 min. Trypsin was neutralised by the addition of 500 μl of AdRPMI 1640 medium + 10% FBS and POS were centrifuged at 300 × *g*. Cell pellets were resuspended in FACS buffer (PBS with 2% FBS) and transferred to FACS tubes. 5 mM DRAQ5 (Biostatus, DR50200; 1:2500) was used to distinguish cells from debris and outer segments. Cells were washed with 0.2% trypan blue solution (Sigma, T8154) to quench fluorescence from bound POS, washed with PBS and suspended in FACS buffer. Samples were analysed immediately on a LSRII flow cytometer and 10,000 events were collected per sample. Results were analysed using FacsDiva software.

### RPE cytokine secretion studies

Medium from basal and apical chambers of transwell inserts were collected from RPE cells of healthy controls and patients. The levels of PEDF and VEGF secretion were measured by using human PEDF-ELISA Kit (Cusabio, CSB-E08818h) and human VEGF-ELISA Kit (Life technologies KHG0111) according to the manufacturer’s instructions.

### RPE characterisation by immunocytochemistry

Cells were fixed in 4% formaldehyde (Sigma, 47608) for 15 min at room temperature and permeabilised with 0.25% Triton X-100 (Sigma, T8787) for 15 min, followed by treatment with blocking solution (3% BSA in PBS, Sigma, A3311) for 30 min at room temperature. Cells were treated with primary antibodies anti-bestrophin (Abcam, ab2182, 1:300), anti-sodium potassium ATPase (Alexa Fluor® 488 conjugate) (Abcam, ab197713, 1:50), pericentrin (Abcam, ab28144, 1:500), MERTK (Bethyl, A300-222A, 1:200), ARL13B (Proteintech, 17711-1-AP, 1:500), collagen IV (Abcam, ab6586, 1:200), PRPF31 (Abnova, PAB7154, 1:500) and SNRPB monoclonal antibody (Y12) (ThermoFisher, MA5-13449, 1:500), overnight at 4 °C, and with secondary antibodies anti-rabbit FITC (Sigma, T9887, 1:500) or anti-mouse FITC (Jackson Immuno Research, 715-095-151, 1:500) and anti-mouse Cy3 (Jackson Immuno Research, 115-165-003, 1:500) or anti-rabbit Cy3 (Jackson Immuno Research, 111-165-003, 1:500) diluted in PBS for 1 h at room temperature. Washes with PBS were carried out between and after treatments. Finally, cells were treated with the nuclear stain-DAPI (Partec, 05-5005), and imaged using a Nikon A1R confocal microscope in combination with the associated NIS Elements software. All antibody details are shown in Supplementary Data 6.

### Immunofluorescence and microscopy of hTERT-RPE1 and RPE

Immunofluorescence staining was performed as described previously^49^. hTERT-RPE1 cells were cultured as described above. Coverslips were seeded with 10^5^ cells and serum starved in OptiMEM for 48 h after transfection to induce ciliogenesis. Twenty-five pmoles of siRNA was reverse-transfected using Lipofectamine RNAiMAx (Invitrogen). Ciliated cells were fixed in ice-cold methanol for 5 min at −20 °C, treated with 0.05% Triton X-100 in PBS for 5 min, and blocked in 1% non-fat milk in PBS for 30 min. Fixed cells were stained for 90 min with appropriate primary antibodies. Coverslips were then washed in PBS and stained for 1 h with appropriate Alexa Fluor-conjugated secondary antibodies (Life Technologies). Coverslips were washed again with PBS before mounting in Prolong Gold anti-fade mountant (Molecular Probes). mIMCD3 cells were seeded at 2.5 × 10^5^ cells/well on sterile glass coverslips in six-well plates and fixed in ice-cold methanol. Cells were blocked in 1% non-fat milk in PBS for 30 min. Images were obtained using a Zeiss ApoTome structured illumination microscope (SIM), equipped with a ×63 objective oil lens, or a Nikon A1R confocal microscope with ×100 oil objective lens controlled by NIS Elements AR 4.20.01 (Nikon) software. Optical sections were generated through structured processing using Axiovision 4.3 (Zeiss) or NIS Elements AR 4.20.01 (Nikon) software. Images were analysed using Adobe Photoshop CS and FIJI software. Images were assembled with Adobe Illustrator CS. All antibody details are shown in Supplementary Data 6.

### Characterisation of retinal organoids by immunocytochemistry

iPSC-derived retinal organoids were fixed in 4% paraformaldehyde at room temperature for 20 min. Post-fixation retinal organoids were incubated overnight with 30% sucrose in PBS, and then frozen and cryosectioned. The frozen sections were stained for a panel of retinal-specific antibodies. Antibodies against the following proteins were used at the indicated dilutions: RECOVERIN (Merck Millipore, AB5585, 1:800), NRL (Santa Cruz, sc-374277, 1:800), CRALBP (Abcam, ab15051, 1:500), HuC/D (ThermoFisher, A21271, 1:500), PKCα (BD Biosciences, 610107, 1:500), ARL13B (Proteintech, 17711-1-AP, 1:500), PRPF31 (Abnova, PAB7154, 1:500), SNRPB monoclonal antibody (Y12) (ThermoFisher, MA5-13449, 1:500). The following secondary antibodies were used: anti-mouse-IgG-FITC (Jackson Immuno Research, 715-095-151, 1:500), anti-mouse-IgG-Cy3 (Jackson Immuno Research/115-165-003, 1:500), anti-rabbit-IgG-Cy3 (Jackson Immuno Research/111-165-003, 1:500), anti-goat-IgG-FITC (Jackson Immuno Research/705-096-147, 1:500). Nuclei were labelled with blue-DAPI (ThermoFisher, 62248). All antibody details are shown in Supplementary Data 6.

### Cilia length and frequency measurements in RPE cells

The length of cilia was measured by a 3D method using Imaris 8.3 Software (Bitplane Inc). Immunocytochemistry Z-stacks images of RPE samples from patients and controls were uploaded into Imaris. Surfaces were created to cover all the length of the cilia across the bottom and top of the stacks. The values were given in μm and a minimum of 150 cilia were independently measured for each sample. The cilia incidence was calculated by counting the number of cilia per cell in each image. Z-stacks were uploaded into Imaris software and spots were created for the blue (DAPI) and green (FITC) channels to cover all nuclei and cilia of cells. The percentage of cilia spots per nuclei spots was calculated and a minimum of 300 cells were counted per sample.

### Cilia length and frequency measurements in retinal organoids

Cryosections from retinal organoids were stained with the ARL13B antibody (Proteintech, 17711-1-AP, 1:500). The images were obtained using Carl Zeiss laser-scanning microscope and Zen software. Maximum intensity projections of z-stacks were used for the analysis. The measurements were performed in MATLAB. The workflow of the method is briefly explained as follows. CZI image files are first imported into MATLAB workspace using the ‘bfopen’ function written by Bio-Formats^50^. This allowed us to access channels individually as grayscale images. Hysteresis thresholding is used to segment the cells and filter out noise from the images^51^. The concept of this method is to use dual thresholding values, such that all the pixels with intensity values above the upper threshold value are first marked as cell pixels, any neighbourhood pixel above the lower threshold that is connected to each of the first pass pixels are also classified as cell pixels. This produces segmentation with fewer isolated points, giving a better result than a simple high-pass thresholding. Segmented objects, such as noise, those sizes smaller than a user selected value are then removed. Finally, a watershed-based method is applied to the binary image to split clustered cells, details of this method can be found in Wang et al.^52^. Because all the pixels of each cell are connected and represented as a single region, an image region property measuring function (regionprops) in MATLAB was used to extract the information of each cell, such as, size, average intensity value and length. The cilia length reported was the average length of all cilia in the image. The frequency of ciliated cells was calculated as cilia numbers/total cell numbers (DAPI) in the same region × 100%.

### Luciferase assay to measure SHH activity

NIH3T3-GL cells (generous gift of Fred Charron, Montreal Clinical Research Institute) were grown in DMEM supplemented with 10% FCS (Sigma-Aldrich, F7524). The cells were passaged at 1:10 ratio twice every week. The cell line was tested for mycoplasma every 3 months. These cells stably express Firefly luciferase under the control of a Gli-response elements and Renilla luciferase constitutively. For the luciferase assay, 20,000 cells were plated into each well of a 96-well plate and two rows each were reverse-transfected with 25 pmoles of siRNA against *PRPF31, Ptch1* and a Scrambled control using Lipofectamine RNAimax. Twenty-four hours later, the cells were treated with 100 nM SAG (Cayman Chemical Company, 11914) or vehicle control for 48 h. The cells were then collected in 1XPLB buffer using the Dual Luciferase Reporter Assay system (Promega, E1910). The assays were run on a Berthold Mithras LB 940 plate reader with dual injector system as per the manufacturer’s protocol. Assay results were expressed as a ratio of firefly: *Renilla* luciferase activities in arbitrary units. The data were analysed by one-way ANOVA followed by Tukey’s multiple comparison test.

### RNA sequencing

Total RNA was extracted from tissue using TRIzol (ThermoFisher Scientific Inc). RNA samples were treated with a TURBO DNA-free™ Kit (Ambion Inc.) using the conditions recommended by the manufacturers, and then cleaned with a RNA Clean & Concentrator™−5 spin column (Zymo Research Corp.). RNA was tested for quality and yield using a NanoDrop 1000 spectrophotometer and an Agilent 2100 Bioanalyzer. RNA-seq analysis was performed for all patients and all controls as triplicate biological repeats in all cell types: fibroblasts, iPSC, iPSC-derived RPE and iPSC-derived retinal organoids. To minimise bubble PCR artefacts, we used 100 ng of purified total RNA in library preparation, following the 'TruSeq' Illumina protocol. In brief, RNA was polyA-selected, chemically fragmented to about 200 nt in size, and cDNA synthesised using random hexamer primers. Each individual library received a unique Illumina barcode. RNA-seq was performed on an Illumina HiSeq 2000 or HiSeq2500 instrument with six or eight libraries multiplexed per flow cell lane using 100 bp paired-end reads. This resulted in an average of 250 million reads per lane, with an average of 40 million reads per sample. Raw reads were aligned to the human (*Homo sapiens*) full genome (GRCm38, UCSC mm10) using STAR, a splice-aware aligner^53^. GTF transcript annotation files were downloaded from Ensembl. Transcripts were assembled using STAR, followed by estimates of raw gene counts using HTSeq^54^. Differential gene expression was analysed using DESeq2^55^ with statistical significance expressed as a *p* value adjusted for a false discovery rate of 0.01 using Benjamini–Hochberg correction for multiple testing.

Alternate splicing analysis was then carried out using rMATS^56^. For each comparison being made, we used the sorted BAM files produced by STAR to run rMATS using default unpaired settings. Reported splicing changes were considered significant if they had a *p* value <0.05 and a change in inclusion-level difference of more than 5%. GO enrichment analysis was carried out on the genes found to have significant splicing changes via clusterProfiler^57^. Multiple testing corrections were carried out using the Benjamini–Hochberg method with an adjusted *p* value <0.05, denoting significantly enriched gene ontology.

### Production of lentiviral particles and transduction of cells

The minigene reporter encoding the adenovirus E1A transcript was subcloned from pMTE1A plasmid^32,33^ into the *Pme*I site of the pWPI lentiviral vector (Addgene; Trono lab). Lentiviral particles were produced in HEK293T cells grown in DMEM medium with 10% FBS. The cells were transfected with pWPI-E1A and the packaging plasmids psPAX2 and pMD2.G (Addgene) using PEIpro transfection reagent (Polyplus transfection). After 54 h, the medium containing lentiviral particles was centrifuged at 1000 × *g* for 5 min and cleared using a 0.45 μm filter. The lentivirus was concentrated using Amicon Ultra 100 kD MWCO centrifugal filter units (Millipore) and aliquots were stored at −80 °C. For lentiviral transduction, cells were seeded in six-well plates with 2 ml medium and infected with the concentrated lentivirus in the presence of 8 µg/ml polybrene (Sigma). After 24 h, the culture medium was refreshed, and 36 h later cells were washed with PBS and harvested.

### E1A alternative splicing assays

Total RNA was extracted from cells transduced with the E1A lentivirus using an RNA extraction kit (Macherey Nagel). E1A alternative splicing was analysed by RT-PCR with 1 µg of the total RNA sample using the high-capacity cDNA reverse transcription kit (Applied Biosystems) and GoTaq DNA polymerase (Promega). PCR was performed with the 5′-end radiolabelled exon 1 forward primer (5′-GTTTTCTCCTCCGAGCCGCTCCGA) and the exon 2 reverse primer (5′-CTCAGGCTCAGGTTCAGACACAGG) by using the following programme: 95 °C for 2 min, 30 cycles of 95 °C for 30 s, 64 °C for 30 s, 72 °C for 1 min, and a final step of 72 °C for 5 min. PCR products were separated by denaturing PAGE, visualised by autoradiography using a Typhoon Trio plus scanner (GE Healthcare) and quantified using Quantity One software (Bio-Rad).

### Western blot analysis

Cells were washed with PBS and lysed in lysis buffer (40 mM HEPES pH 7.4, 150 mM NaCl, 1% Triton X-100, 1 mM phenylmethylsulfonylfluoride, 1 mM sodium orthovanadate and 0.5 mM DTT) supplemented with phosphatase inhibitor and EDTA-free protease inhibitor cocktails (Roche). The concentration of total protein in cleared lysates was measured by Bradford assay and about 20 µg of each sample was analysed by western blotting followed by immunostaining using antibodies against SART1, PRPF8, Snu114, PRPF31 (against its N terminus or C terminus), PRPF4 and SF3b155, and the Amersham ECL detection kit (GE Healthcare). All antibody details are shown in Supplementary Data 6. Uncropped blots are shown in Supplementary Figure 11.

### Analysis of snRNP levels by glycerol gradient fractionation

Nuclear extracts (200 µg each) were diluted with an equal volume of gradient buffer (G150: 20 mM HEPES pH 7.9, 150 Mm NaCl, 1.5 mM MgCl_2_ and 0.5 mM DTT) and sedimented on linear 4 ml 10-30% (v/v) glycerol gradients in the G150 buffer. After ultracentrifugation in a Sorvall TH-660 rotor for 14 h at 29,000 rpm (114,000 × *g*), the gradients were separated into 24 fractions. To analyse the relative levels of snRNPs in the nuclear extracts, proteins in the gradient fractions were digested by Proteinase K in 20 mM HEPES pH 7.9, 150 mMNaCl, 10 mM EDTA, 1% (w/v) SDS for 45 min at 42 °C, the RNAs were extracted by phenol/chloroform/isoamylalcohol and precipitated. The isolated RNAs were separated by denaturing 8% urea PAGE followed by Northern blotting using 5′-end radiolabeled DNA probes against U1, U2, U4, U6 and U5 snRNAs. To analyse the association of selected splicing proteins with the tri-snRNP, proteins were precipitated from gradient fractions and separated on NuPAGE 4−12% Bis–Tris gels (Invitrogen) followed by blotting and immunostaining using antibodies against PRPF8, Brr2, Snu114, PRPF31 (against its C terminus), PRPF4 and SF3b155, and the Amersham ECL detection kit (GE Healthcare). All antibody details are shown in Supplementary Data 6.

### TMT labelling for mass spectrometry

Total cell lysates were prepared from 1 million RP11VS retinal organoid or RPE cells and the corresponding Cas9-corrected cells according to the protocol described for Pierce Mass Spec Sample Prep Kit (Thermo Scientific). Lysates were diluted to 130 µl and sonicated using Covaris S220 ultrasonicator (Covaris). Protein concentrations were determined using the Pierce BCA protein assay kit and 100 µg of the total proteins from patient or Cas9-corrected control cells were processed for isobaric tandem mass tag (TMT) labelling using TMTduplex Isobaric Mass Tagging Kit (Thermo Scientific) according to the manufacturer’s instructions. Briefly, samples were reduced by the addition of TCEP, alkylated with iodoacetamide and acetone precipitated. Protein pellets were resuspended in 50 mM TEAB (triethyl ammonium bicarbonate) buffer followed by digestion with trypsin overnight at 37 °C. The patient and Cas9-corrected control samples were, respectively, labelled with TMT-127 and TMT-126 reagents for 1 h at room temperature and the reactions were quenched by 5% hydroxylamine for 15 min. Next, 50 µg of TMT-labelled peptides from patient and control cells were combined and cleaned up using C18 spin columns (Harvard Apparatus). The samples were dried down by SpeedVac (Eppendorf) and reconstituted in 100 µl buffer A (10 mM NH_4_OH). Fifty microliters of peptide mixtures were separated in 80 fractions on an XBridge BEH C18 HPLC column (150 mm × 1 mm ID, 3.5 µm; Waters) using a gradient of buffer B (10 mM NH_4_OH, 80% acetonitrile) over 90 min. The elution fractions were combined to 20 fractions, dried down by SpeedVac and resuspended in 20 µl of 0.1% trifluoroacetic acid (TFA).

### LC/MS/MS analysis

Peptides in each fraction were analysed in three replicates on either an Orbitrap Fusion or a Q Exactive HF-X mass spectrometer (Thermo Fisher Scientific), both of which are coupled with an UltiMate 3000 RSLCnano HPLC system (Thermo Fisher Scientific). First, the peptides were desalted on a reverse phase C18 pre-column (Dionex 5×, 0.3 mm ID) for 3 min. After 3 min, the pre-column was switched online with the analytical column (30 cm long, 75 μm ID) prepared in-house using ReproSil-Pur C18 AQ 1.9 μm reversed phase resin (Dr. Maisch GmbH). Solvent A consisted of 0.1% formic acid in H_2_O, and B consisted of 80% acetonitrile and 0.1% formic acid in H_2_O. The peptides were eluted with buffer B (8–42% gradient) at a flow rate of 300 nL/min over 70 min. The pre-column and the column temperature were set to 50 °C during chromatography. On the Orbitrap Fusion, a data-dependent synchronous precursor selection (SPS)-based MS3 method was used, where the most intense precursors in the *m/z* range of 400–1200 Th and with the charge state of 2–7 were selected from a survey MS1 scan for MS2 fragmentation with an isolation window of 0.7 Th. Subsequently, up to 10 resulting MS2 top fragments were further collected simultaneously with an isolation window of 1.6 Th for MS3 analysis. A maximal cycle time of 4 s was maintained at all time. MS1 scans were acquired at a resolution of 120,000 and an AGC target of 2E5. Selected precursors underwent CID fragmentation with normalised collision energy (NCE) of 35. MS2 scans were acquired in the ion trap in turbo scan mode with a maximum ion injection (IT) time of 50 ms and an AGC target of 1E4. MS3 scans were analysed using HCD fragmentation at NCE of 65 and detected in Orbitrap with a resolution of 30,000. For samples measured on the Q Exactive HF-X, the mass spectrometer was operated in Top20 data-dependent mode, where the most intense 20 precursors in the *m/z* range of 350–1600 Th were selected for MS2 fragmentation with an isolation window of 0.8 and NCE of 32. MS2 spectra were acquired in Orbitrap with a resolution of 15,000 and a maximum IT of 64 ms. AGC for MS1 and MS2 scans were 3E6 and 1E5, respectively. Dynamic exclusion (DE) was set to 20 s for both instruments.

### Data processing

MS/MS spectra were searched against a Swiss-Prot human database containing 20,341 reviewed protein entries using Mascot algorithm (Matrix Science) via Proteome Discoverer 2.2 (PD, Thermo Fisher Scientific). Precursor and fragment ion mass tolerances were, respectively, set to 10 ppm and 0.6 Dalton for raw files from Orbitrap Fusion or 10 ppm and 0.02 Dalton for the ones from Q Exactive HF-X after initial recalibration. Protein N-terminal acetylation, methionine oxidation and glutamine/asparagine deamidation were allowed as variable modifications. Cysteine carbamidomethylation and TMT2plex on both peptide N terminus and lysine residue were defined as fixed modifications. Minimal peptide length was set to six amino acids, with a maximum of two missed cleavages. Mascot percolator was applied to improve the accuracy and sensitivity of peptide identification. The false discovery rate (FDR) was set to 1% at both the peptide spectrum match (PSM) level and the protein level, respectively, using Mascot Percolator and a built-in Protein FDR Validator node in PD. Quantitative measurement was based on relative abundance of the detected TMT reporter ions in MS3 or MS2 spectra for raw files from the Orbitrap Fusion or Q Exactive HF-X, respectively. At least two quantifiable unique peptides in each replicate were required for protein quantification. Protein ratios were log transformed and then median normalised based on the assumption that the majority of the proteins are unaffected. The reported RP11VS/Cas9-RP11VS ratios are the average of at least two replicates. To identify the differentially regulated proteins, the corresponding Z scores were calculated and those proteins with Z scores less than −1.5 or greater than +1.5 were defined as regulated. 1D annotation enrichment analysis was carried out by the Perseus software version 1.6.1.3 with a Benjamini–Hochberg FDR 2%^58^.

### Quantification and statistical analysis

*P* values were calculated of normally distributed data sets using a two-tailed Student’s *t* test, or one-way ANOVA with Dunnett’s post hoc test, or two-way ANOVA with Bonferroni post hoc tests using GraphPad Prism Software Inc. (San Diego, CA, USA). Statistical analyses represent the mean of at least three independent experiments, error bars represent standard error of mean (s.e.m.) or as otherwise indicated. The statistical significance of pairwise comparisons shown on bar graphs is indicated by n.s. not significant, *p < 0.05, **p < 0.01, ***p < 0.001 and ****p < 0.0001. For cell populations, a minimum of 100 cells were counted from >10 separate fields of view.

## Electronic supplementary material

Supplementary Information

Description of Additional Supplementary Files

Supplementary Data 1

Supplementary Data 2

Supplementary Data 3

Supplementary Data 4

Supplementary Data 5

Supplementary Data 6

Supplementary Data 7

Supplementary Data 8

Supplementary Data 9

## Data Availability

The trimmed FASTQ data for all human samples were uploaded to the European Nucleotide Archive under the accession number PRJEB22885 (human) and PRJNA417002 (mouse). The mass spectrometry proteomics data have been deposited to the ProteomeXchange Consortium via the PRIDE^59^ partner repository with the dataset identifier PXD010821.

## References

[1] Hartong DT, Berson EL, Dryja TP (2006). Retinitis pigmentosa. Lancet.

[2] McKie AB (2001). Mutations in the pre-mRNA splicing factor gene PRPC8 in autosomal dominant retinitis pigmentosa (RP13). Hum. Mol. Genet..

[3] Vithana EN (2001). A human homolog of yeast pre-mRNA splicing gene, PRP31, underlies autosomal dominant retinitis pigmentosa on chromosome 19q13.4 (RP11). Mol. Cell.

[4] Chakarova CF (2002). Mutations in HPRP3, a third member of pre-mRNA splicing factor genes, implicated in autosomal dominant retinitis pigmentosa. Hum. Mol. Genet..

[5] Zhao C (2009). Autosomal-dominant retinitis pigmentosa caused by a mutation in SNRNP200, a gene required for unwinding of U4/U6 snRNAs. Am. J. Hum. Genet..

[6] Keen TJ (2002). Mutations in a protein target of the Pim-1 kinase associated with the RP9 form of autosomal dominant retinitis pigmentosa. Eur. J. Hum. Genet..

[7] Maita H (2000). PAP-1, a novel target protein of phosphorylation by pim-1 kinase. Eur. J. Biochem..

[8] Sullivan LS (2006). Prevalence of disease-causing mutations in families with autosomal dominant retinitis pigmentosa: a screen of known genes in 200 families. Invest. Ophthalmol. Vis. Sci..

[9] Chen X (2014). PRPF4 mutations cause autosomal dominant retinitis pigmentosa. Hum. Mol. Genet..

[10] Tanackovic G (2011). A missense mutation in PRPF6 causes impairment of pre-mRNA splicing and autosomal-dominant retinitis pigmentosa. Am. J. Hum. Genet..

[11] Ezquerra-Inchausti M (2017). High prevalence of mutations affecting the splicing process in a Spanish cohort with autosomal dominant retinitis pigmentosa. Sci. Rep..

[12] Murphy D, Cieply B, Carstens R, Ramamurthy V, Stoilov P (2016). The Musashi 1 controls the splicing of photoreceptor-specific exons in the vertebrate retina. PLoS Genet..

[13] May-Simera HL (2018). Primary cilium-mediated retinal pigment epithelium maturation is disrupted in ciliopathy patient cells. Cell Rep..

[14] Tanackovic G, Rivolta C (2009). PRPF31 alternative splicing and expression in human retina. Ophthalmic Genet..

[15] Tanackovic G (2011). PRPF mutations are associated with generalized defects in spliceosome formation and pre-mRNA splicing in patients with retinitis pigmentosa. Hum. Mol. Genet..

[16] Ivings L (2008). Evaluation of splicing efficiency in lymphoblastoid cell lines from patients with splicing-factor retinitis pigmentosa. Mol. Vis..

[17] Comitato A (2007). Mutations in splicing factor PRPF3, causing retinal degeneration, form detrimental aggregates in photoreceptor cells. Hum. Mol. Genet..

[18] Bujakowska K (2009). Study of gene-targeted mouse models of splicing factor gene Prpf31 implicated in human autosomal dominant retinitis pigmentosa (RP). Invest. Ophthalmol. Vis. Sci..

[19] Graziotto JJ, Inglehearn CF, Pack MA, Pierce EA (2008). Decreased levels of the RNA splicing factor Prpf3 in mice and zebrafish do not cause photoreceptor degeneration. Invest. Ophthalmol. Vis. Sci..

[20] Graziotto JJ (2011). Three gene-targeted mouse models of RNA splicing factor RP show late-onset RPE and retinal degeneration. Invest. Ophthalmol. Vis. Sci..

[21] Farkas MH (2014). Mutations in pre-mRNA processing factors 3, 8, and 31 cause dysfunction of the retinal pigment epithelium. Am. J. Pathol..

[22] Carr AJ (2009). Protective effects of human iPS-derived retinal pigment epithelium cell transplantation in the retinal dystrophic rat. PLoS ONE.

[23] Mellough CB (2015). IGF-1 signaling plays an important role in the formation of three-dimensional laminated neural retina and other ocular structures from human embryonic stem cells. Stem Cells.

[24] Kuwahara A (2015). Generation of a ciliary margin-like stem cell niche from self-organizing human retinal tissue. Nat. Commun..

[25] Wilmes A (2017). Towards optimisation of induced pluripotent cell culture: Extracellular acidification results in growth arrest of iPSC prior to nutrient exhaustion. Toxicol. Vitr..

[26] Melguizo-Sanchis D (2018). iPSC modeling of severe aplastic anemia reveals impaired differentiation and telomere shortening in blood progenitors. Cell Death Dis..

[27] Karakousis PC (2001). Localization of pigment epithelium derived factor (PEDF) in developing and adult human ocular tissues. Mol. Vis..

[28] Kozulin P, Natoli R, Bumsted O’Brien KM, Madigan MC, Provis JM (2010). The cellular expression of antiangiogenic factors in fetal primate macula. Invest. Ophthalmol. Vis. Sci..

[29] Becerra SP (2004). Pigment epithelium-derived factor in the monkey retinal pigment epithelium and interphotoreceptor matrix: apical secretion and distribution. Exp. Eye Res..

[30] Volpert KN, Tombran-Tink J, Barnstable C, Layer PG (2009). PEDF and GDNF are key regulators of photoreceptor development and retinal neurogenesis in reaggregates from chick embryonic retina. J. Ocul. Biol. Dis. Info..

[31] Saint-Geniez M (2008). Endogenous VEGF is required for visual function: evidence for a survival role on muller cells and photoreceptors. PLoS ONE.

[32] Caceres JF, Stamm S, Helfman DM, Krainer AR (1994). Regulation of alternative splicing in vivo by overexpression of antagonistic splicing factors. Science.

[33] Zerler B (1986). Adenovirus E1A coding sequences that enable ras and pmt oncogenes to transform cultured primary cells. Mol. Cell. Biol..

[34] Wheway G (2015). An siRNA-based functional genomics screen for the identification of regulators of ciliogenesis and ciliopathy genes. Nat. Cell Biol..

[35] Kurtovic-Kozaric A (2015). PRPF8 defects cause missplicing in myeloid malignancies. Leukemia.

[36] Liang D (2017). The output of protein-coding genes shifts to circular RNAs when the pre-mRNA processing machinery is limiting. Mol. Cell.

[37] Rose AM (2016). Transcriptional regulation of PRPF31 gene expression by MSR1 repeat elements causes incomplete penetrance in retinitis pigmentosa. Sci. Rep..

[38] Venturini G, Rose AM, Shah AZ, Bhattacharya SS, Rivolta C (2012). CNOT3 is a modifier of PRPF31 mutations in retinitis pigmentosa with incomplete penetrance. PLoS Genet..

[39] Zhu J (2017). Gene and mutation independent therapy via CRISPR-Cas9 mediated cellular reprogramming in rod photoreceptors. Cell Res..

[40] Yu W, Wu Z (2018). In vivo applications of CRISPR-based genome editing in the retina. Front. Cell. Dev. Biol..

[41] Ovando-Roche P, Georgiadis A, Smith AJ, Pearson RA, Ali RR (2017). Harnessing the potential of human pluripotent stem cells and gene editing for the treatment of retinal degeneration. Curr. Stem Cell Rep..

[42] Dong B (2013). Two novel PRP31 premessenger ribonucleic acid processing factor 31 homolog mutations including a complex insertion-deletion identified in Chinese families with retinitis pigmentosa. Mol. Vis..

[43] Singh R (2013). Functional analysis of serially expanded human iPS cell-derived RPE cultures. Invest. Ophthalmol. Vis. Sci..

[44] Kokkinaki M, Sahibzada N, Golestaneh N (2011). Human induced pluripotent stem-derived retinal pigment epithelium (RPE) cells exhibit ion transport, membrane potential, polarized vascular endothelial growth factor secretion, and gene expression pattern similar to native RPE. Stem Cells.

[45] Muthmann JO (2015). Spike detection for large neural populations using high density multielectrode arrays. Front. Neuroinform..

[46] Hilgen G (2017). Pan-retinal characterisation of light responses from ganglion cells in the developing mouse retina. Sci. Rep..

[47] Ran FA (2013). Genome engineering using the CRISPR-Cas9 system. Nat. Protoc..

[48] Deerinck TJ (2010). Enhancing serial block-face scanning electron microscopy to enable high resolution 3-d nanohistology of cells and tissues. Microsc. Microanal..

[49] Dawe HR (2009). Nesprin-2 interacts with meckelin and mediates ciliogenesis via remodelling of the actin cytoskeleton. J. Cell. Sci..

[50] Linkert M (2010). Metadata matters: access to image data in the real world. J. Cell. Biol..

[51] Zimmer C, Labruyere E, Meas-Yedid V, Guillen N, Olivo-Marin JC (2002). Segmentation and tracking of migrating cells in videomicroscopy with parametric active contours: a tool for cell-based drug testing. IEEE Trans. Med. Imaging.

[52] Wang Y, Zhang Z, Wang H, Bi S (2015). Segmentation of the clustered cells with optimized boundary detection in negative phase contrast images. PLoS ONE.

[53] Dobin Alexander, Davis Carrie A., Schlesinger Felix, Drenkow Jorg, Zaleski Chris, Jha Sonali, Batut Philippe, Chaisson Mark, Gingeras Thomas R. (2012). STAR: ultrafast universal RNA-seq aligner. Bioinformatics.

[54] Anders S, Pyl PT, Huber W (2014). HTSeq — a Python framework to work with high-throughput sequencing data. Bioinformatics Online.

[55] Love MI, Huber W, Anders S (2014). Moderated estimation of fold change and dispersion for RNA-seq data with DESeq2. Genome Biol..

[56] Park JW, Tokheim C, Shen S, Xing Y (2013). Identifying differential alternative splicing events from RNA sequencing data using RNASeq-MATS. Methods Mol. Biol..

[57] Yu G, Wang L, Han Y, He Q (2012). clusterProfiler: an R package for comparing biological themes among gene clusters. OMICS.

[58] Tyanova S (2016). The Perseus computational platform for comprehensive analysis of (prote)omics data. Nat. Methods.

[59] Vizcaíno JA (2016). 2016 update of the PRIDE database and related tools. Nucleic Acids Res..

