# Supplementary material for: Disrupted alternative splicing for genes implicated in splicing and ciliogenesis causes PRPF31 retinitis pigmentosa
Source: Nat Commun. 2018 Oct 12;9:4234. doi: 10.1038/s41467-018-06448-y (PMC6185938; doi:10.1038/s41467-018-06448-y)
Supplement: Supplementary file 1 — Supplementary Information [file 41467_2018_6448_MOESM1_ESM.pdf]

<u>Supplementary Information</u>

# Disrupted alternative splicing for genes implicated in splicing and ciliogenesis causes PRPF31 retinitis pigmentosa

Buskin, Zhu, Chichagova, Basu, Mozaffari-Jovin et al.

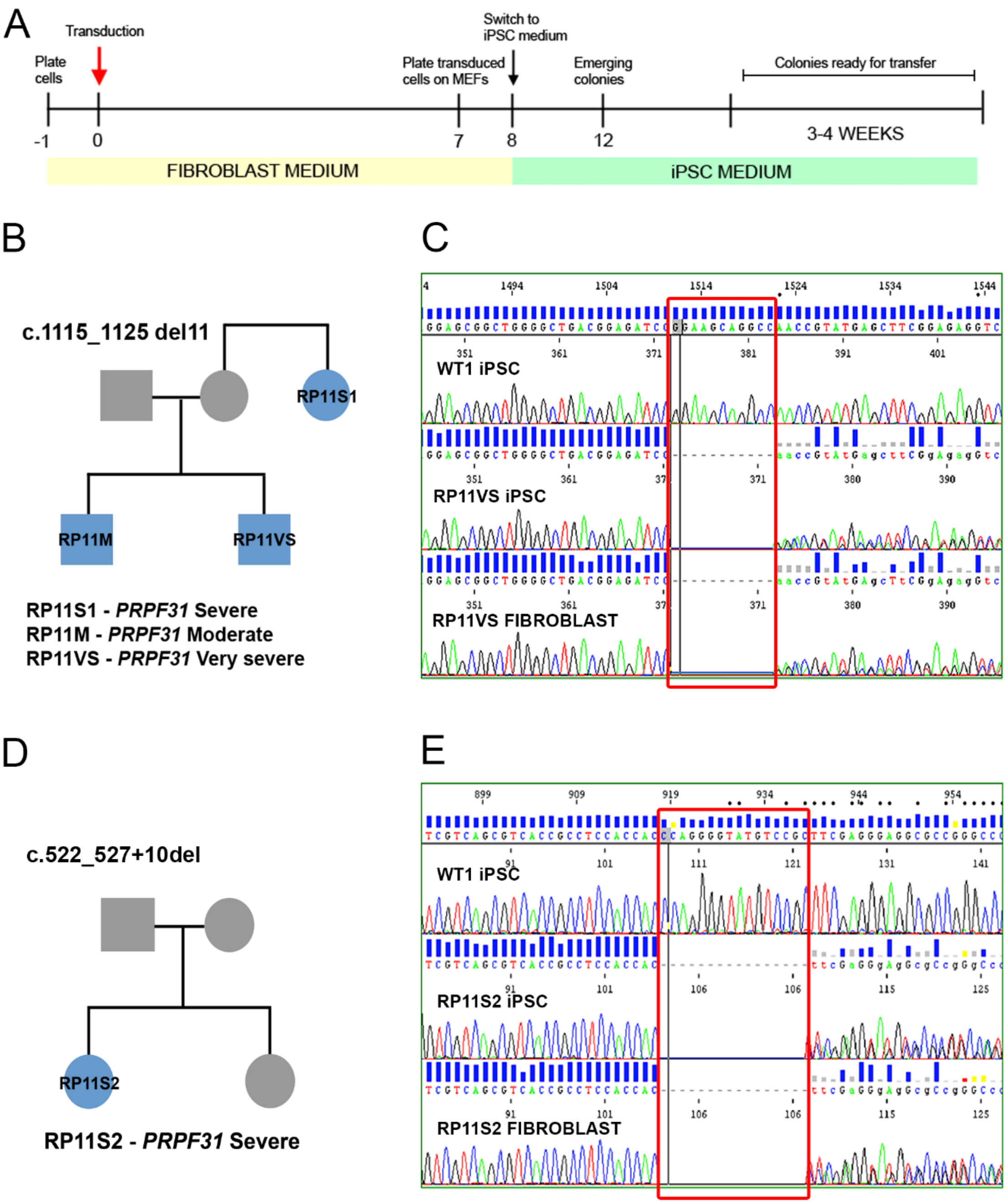

**Supplementary Figure 1. Genotype of RP patients and corresponding iPSCs.** (**A**) iPSC generation timeline; (**B**) Pedigree and phenotype of the patients with c.1115_1125del11 and mutation confirmation (**C**); (**D**) Pedigree and phenotype of the patient with c.522_527+10del and mutation confirmation (**E**).

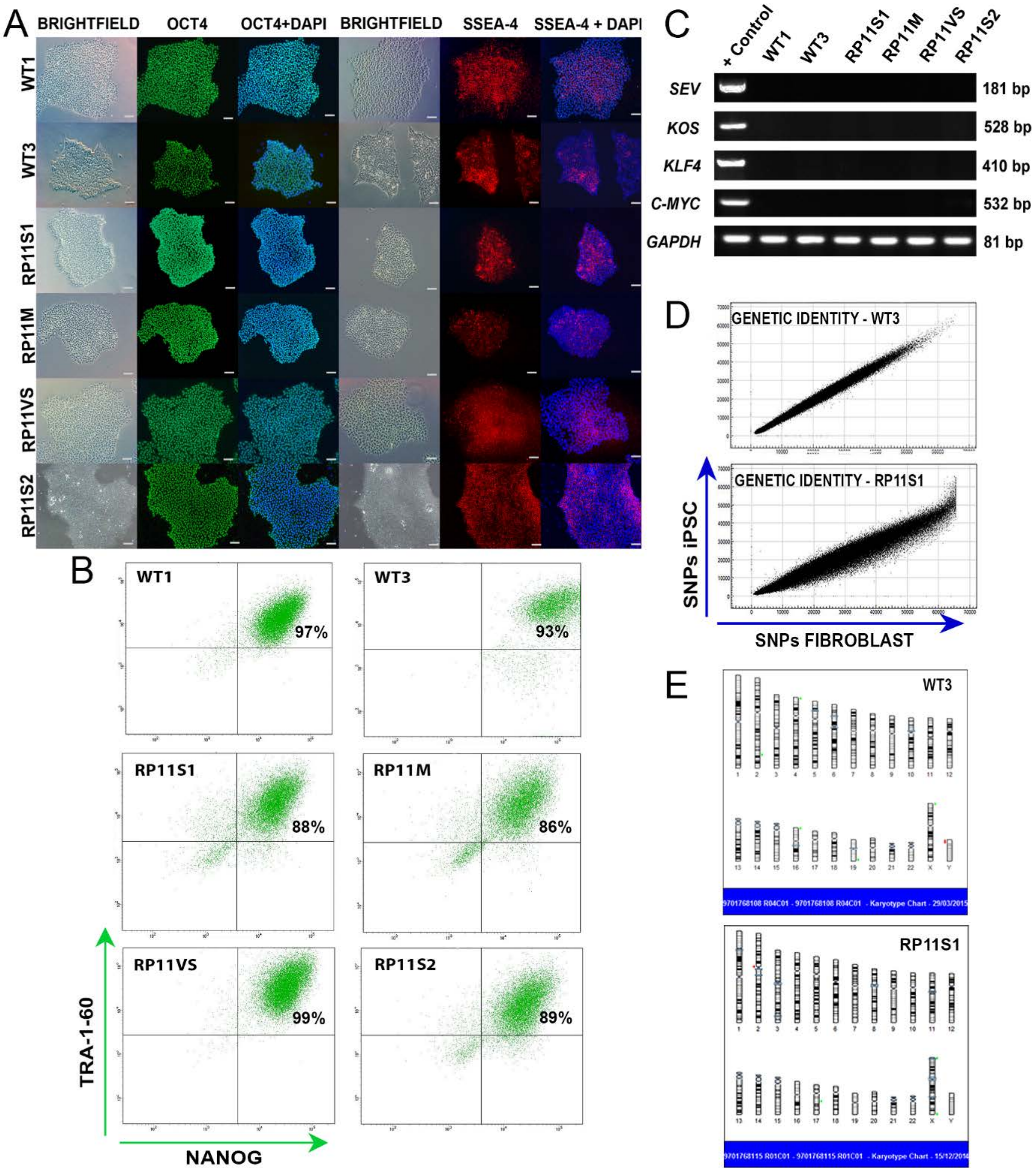

**Supplementary Figure 2. Generation and characterisation of patient and control iPSCs.** (**A, B**) Bright field images, immunostaining and flow cytometric analysis for pluripotency-associated markers (scale bar 50 µm); (**C**) Verification of Sendai virus genome (*SEV*) and the transgenes (*KOS, KLF4, C-MYC*) clearance by RT-PCR; (**D**) Representative CytoSNP analysis confirming the genetic identity between iPSC and parent fibroblast and lack of genomic abnormalities (**E**). A-E: Representative examples from three independent experiments are shown.

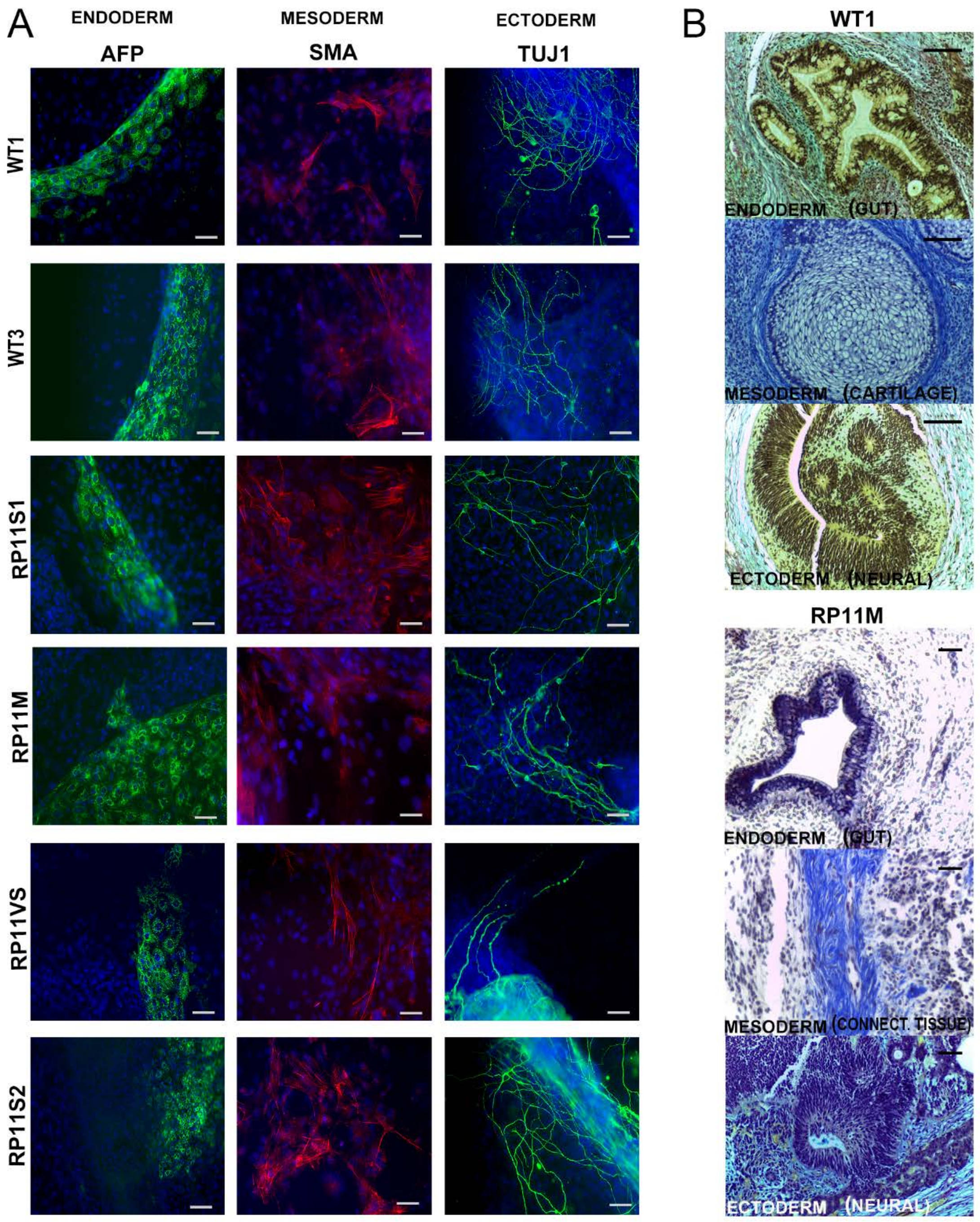

**Supplementary Figure 3. iPSCs were capable of spontaneous differentiation towards cells representative of three germ layers *in vitro*** (scale bar 50 µm) (**A**) **and *in vivo*** (scale bar 100 µm top three panels, 50 µm bottom three panes) **(B).**

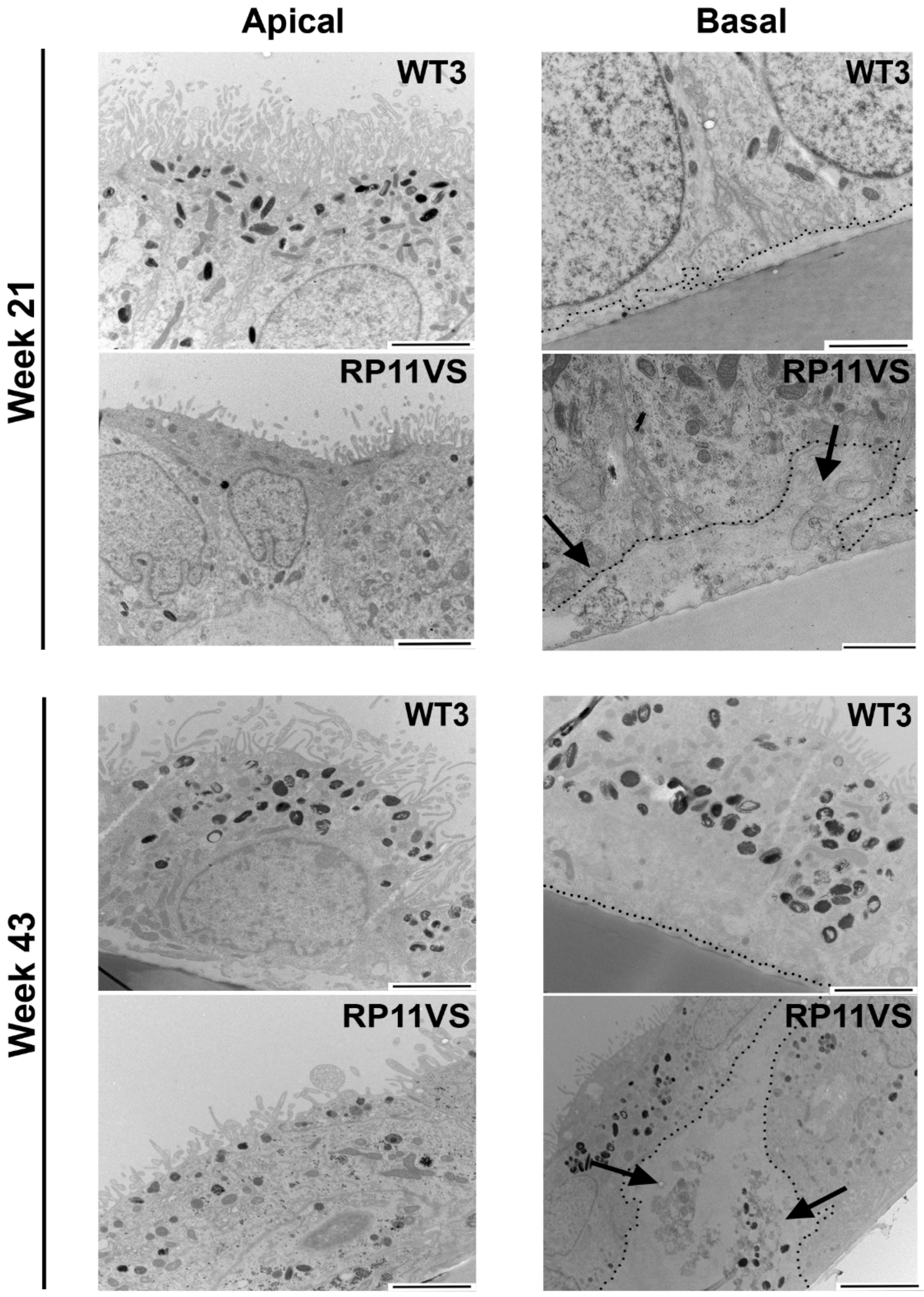


**Supplementary Figure 4. Abnormal ultrastructural features of patient RPE cells.** TEM at 21 and 43 weeks in culture shows shorter and fewer microvilli in RP11 RPE cells and large basal

deposits that were not observed in controls. Examples are representative of at least three independent experiments. Scale bar 2 µm.

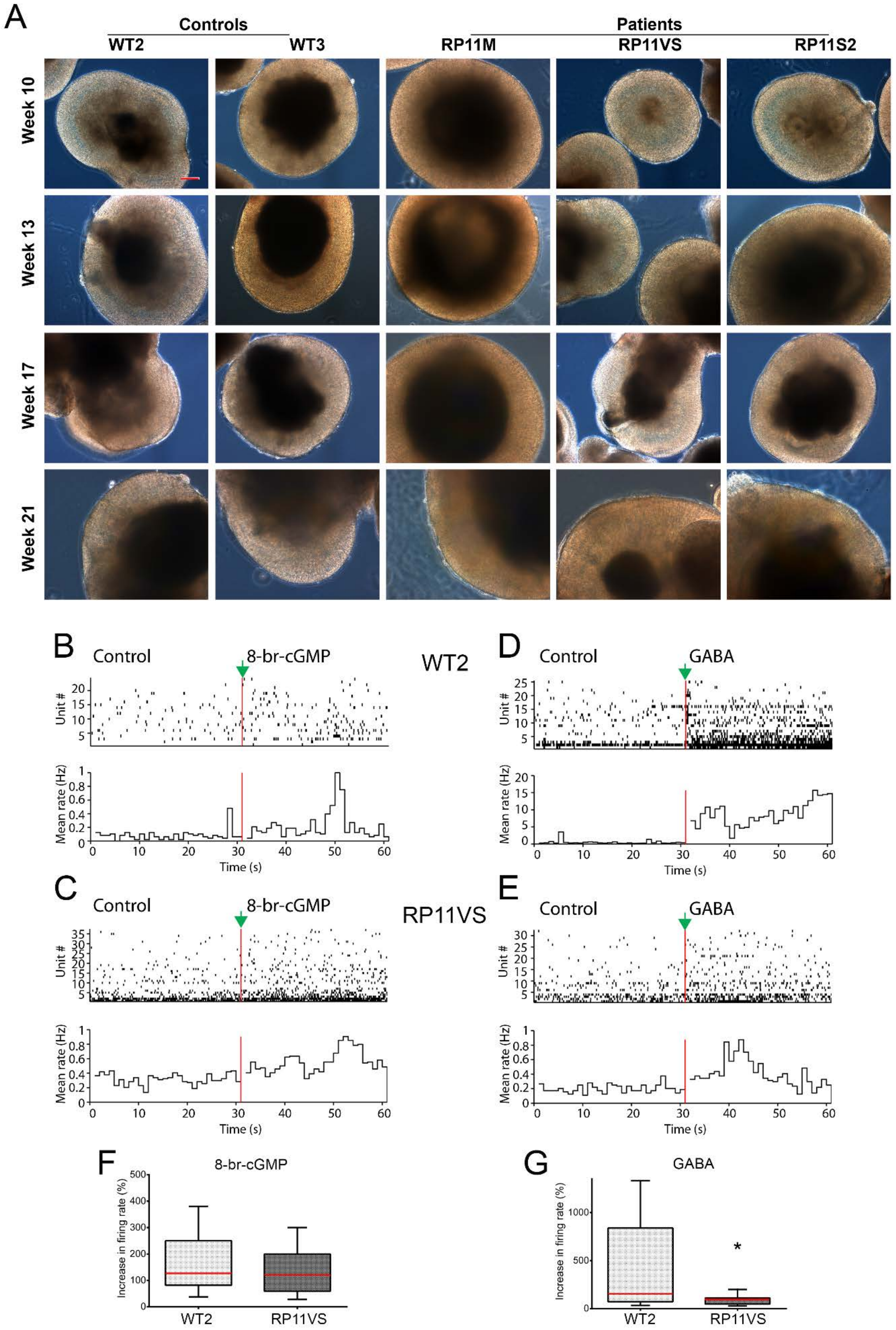


**Supplementary Figure 5. Spiking activity recorded from presumed retinal ganglion cells in 3D retinae derived from control (WT2) and diseased iPSC line (RP11VS) at week 21 of**

**differentiation.** The activity was recorded with a 4096 channel MEA in contact with the tissue. (**A**) Brightfield images of developing retinal neuroepithelium over time: these are representative examples of at least eight independent experiments (scale bar 100 µm); (**B**) Raster plot (top panel) and firing rate histogram (bottom panel) illustrate all the units in a WT2 sample showing an increase in firing in the presence of 8-br-cGMP. In the raster plot, each small vertical bar indicates the time stamp of an action potential. Each row represents a different RGC. The left half illustrates the activity in control conditions. The drug was puffed in the recording chamber (final concentration, 100 µM) at the time indicated by the green arrow and red line; (**C**) Same as in (B) but in a RP sample. The increase in activity is less conspicuous than in (B); (**D**) Same as in (B), but this time in the presence of the neurotransmitter GABA puffed as indicated by the green arrow and red line (final concentration 125µM). Some cells exhibit a strong increase in firing when exposed to GABA in this WT2 sample; (**E**) Same as (D) but in a RP sample. The increase in activity is less conspicuous than in (D); (**F**) Box plot summarising cGMP-mediated increases in firing rate (expressed as % increase from control conditions) in WT2 and RP samples. The box plot shows the median (red line) and interquartile ranges with Tukey whiskers. There is no significant difference between WT2 and RP11VS (Mann Whitney test; N=20 for WT2 and N=37 for RP); (**G**) Same as (F) but for GABA. Here there is significantly less response in RP11VS samples than in WT2 (*, $p<0.02$; Mann Whitney test; N=24 for WT2 and N=26 for RP).

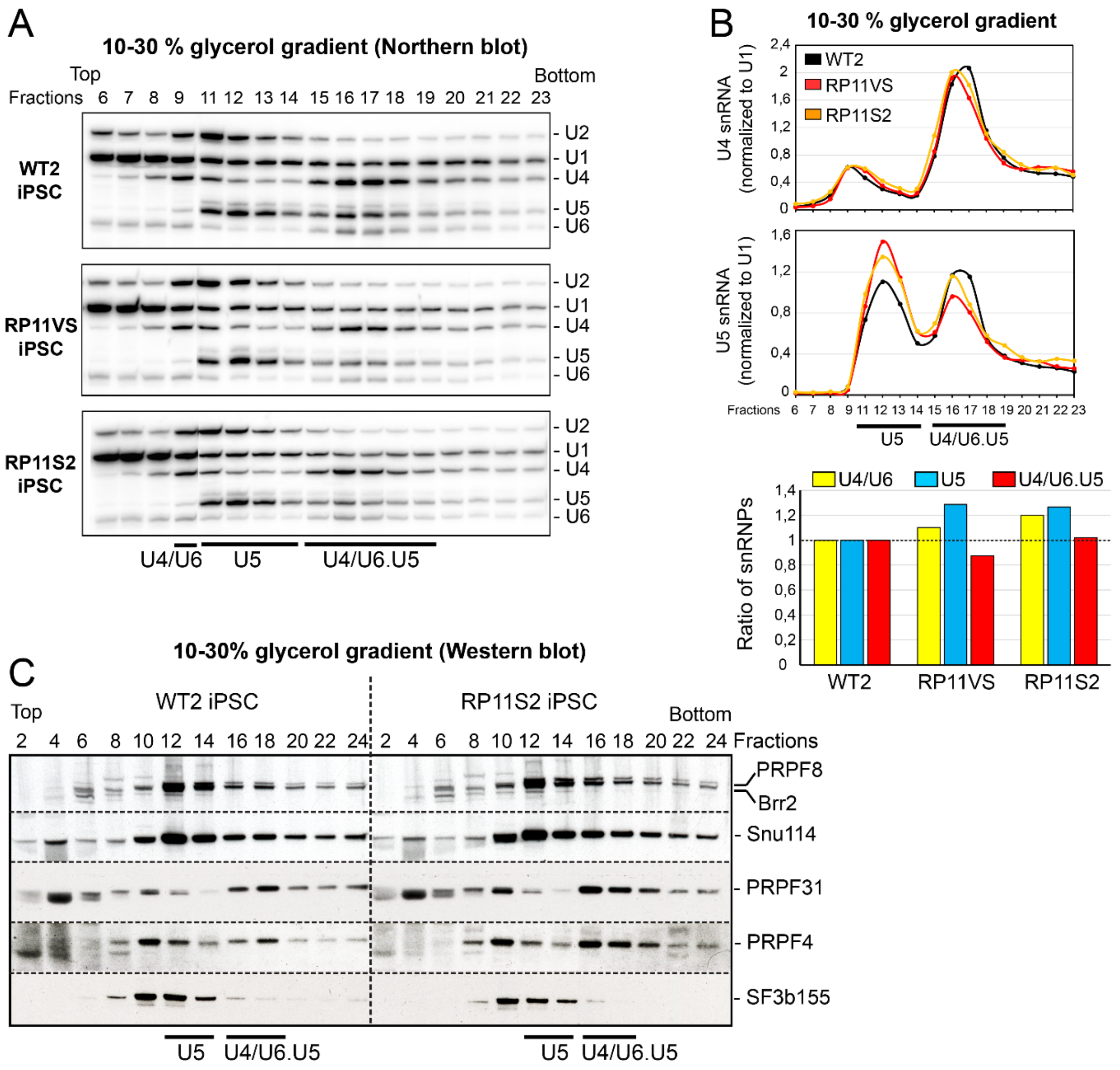


**Supplementary Figure 6. Analysis of snRNP levels in RP11 and control iPSC.** (**A**) Northern blots of glycerol gradient fractions from normal and patient-specific iPSC nuclear extracts; (**B**) Top: Quantification of the U4 and U6 snRNP distributions across the gradient shown in A. Bottom: Ratios of U4/U6, U5 and U4/U6.U5 tri-snRNP in iPSCs quantified from data in A; (**C**) Western blots of even-numbered glycerol gradient fractions of nuclear extracts from wild type and mutant PRPF31 iPSCs. Migration positions of snRNPs are indicated below the gel and the detected proteins at the right. Shown examples are representative of at least 2 independent experiments.

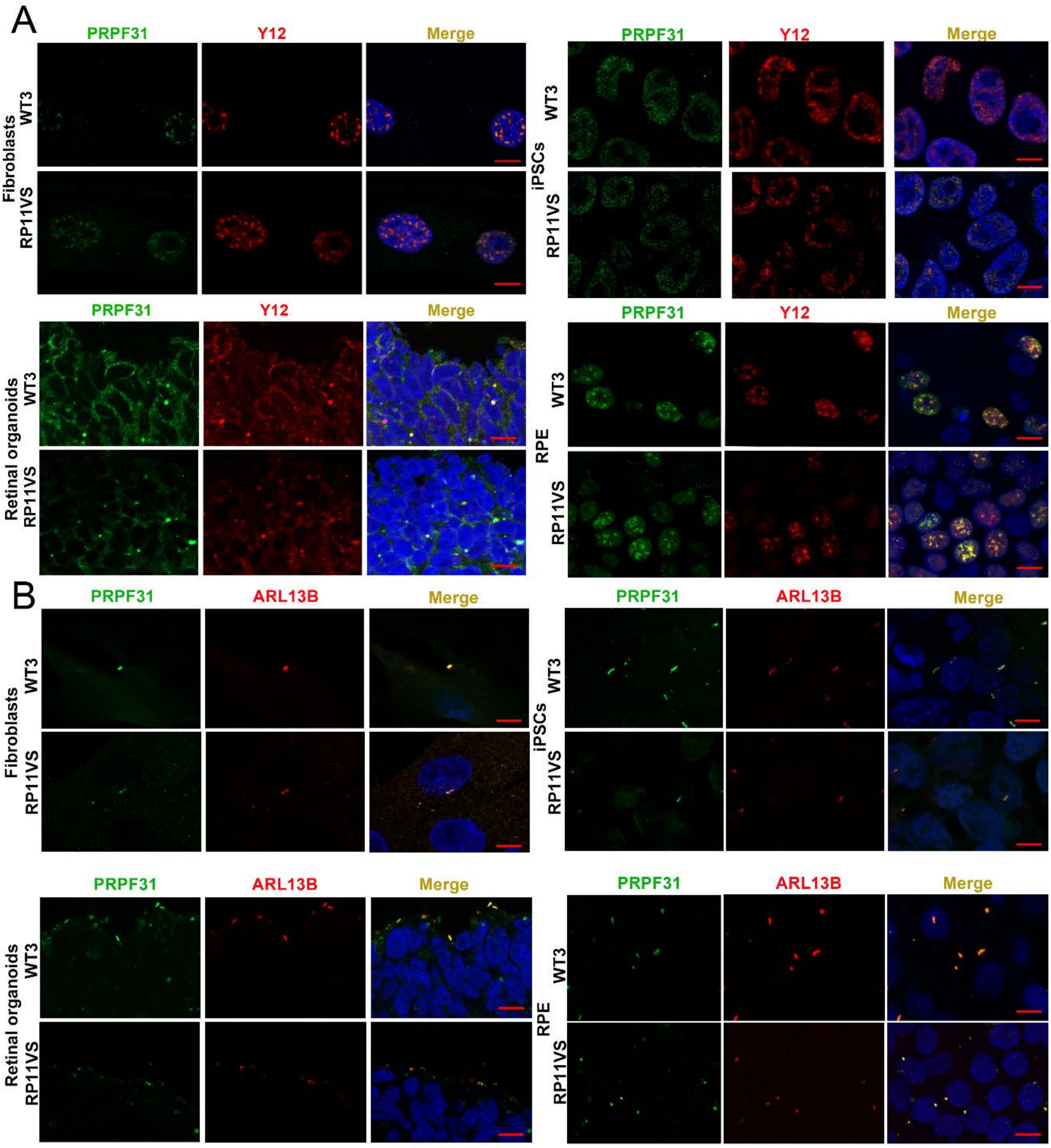


**Supplementary Figure 7. Co-Localisation of PRPF31 with spliceosome component (Y12) (A) and cilia (ARL13B) (B) in fibroblasts, iPSCs, RPE, and retinal organoids.** These are representative examples of at least three independent experiments. Scale bar 100 µm.

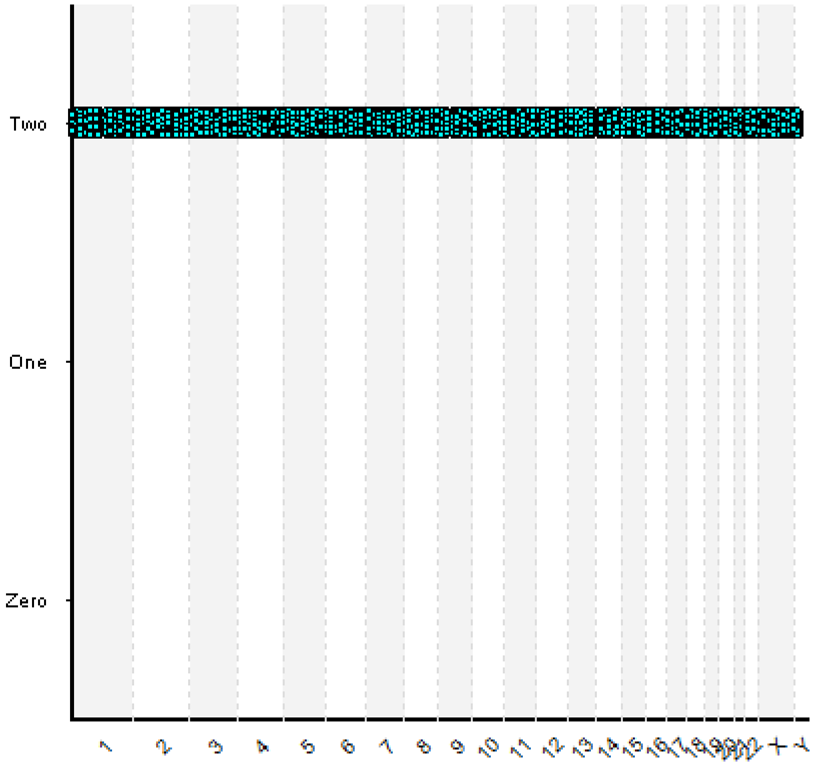


**Supplementary Figure 8. Graph from BlueFuse Multi software version 4.4 comparing the SNP allele between RP11VS and Cas9-RP11VS iSPC showing that all alleles across the whole genome are the same and there are no added genomic instabilities during the Crispr/Cas9 gene editing.**

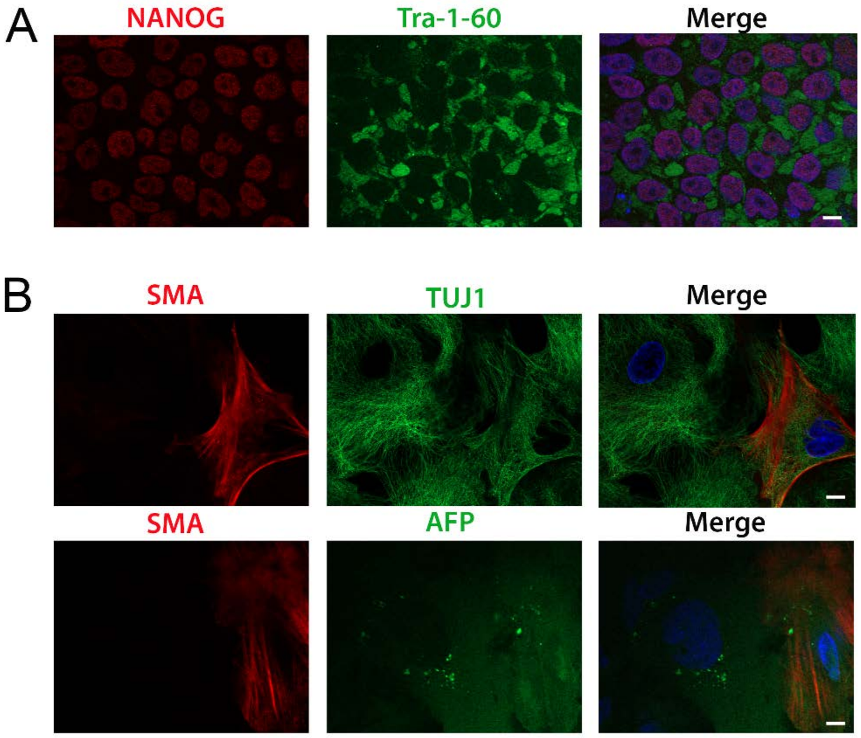


**Supplementary Figure 9. Characterisation of CRISPR/Cas9 iPSCs. (A)** Expression of pluripotency-associated markers NANOG and Tra-1-60; **(B)** CRISPR/Cas9 iPSCs were capable of spontaneous differentiation towards cells representative of three germ layers *in vitro*. Scale bar 10 µm.

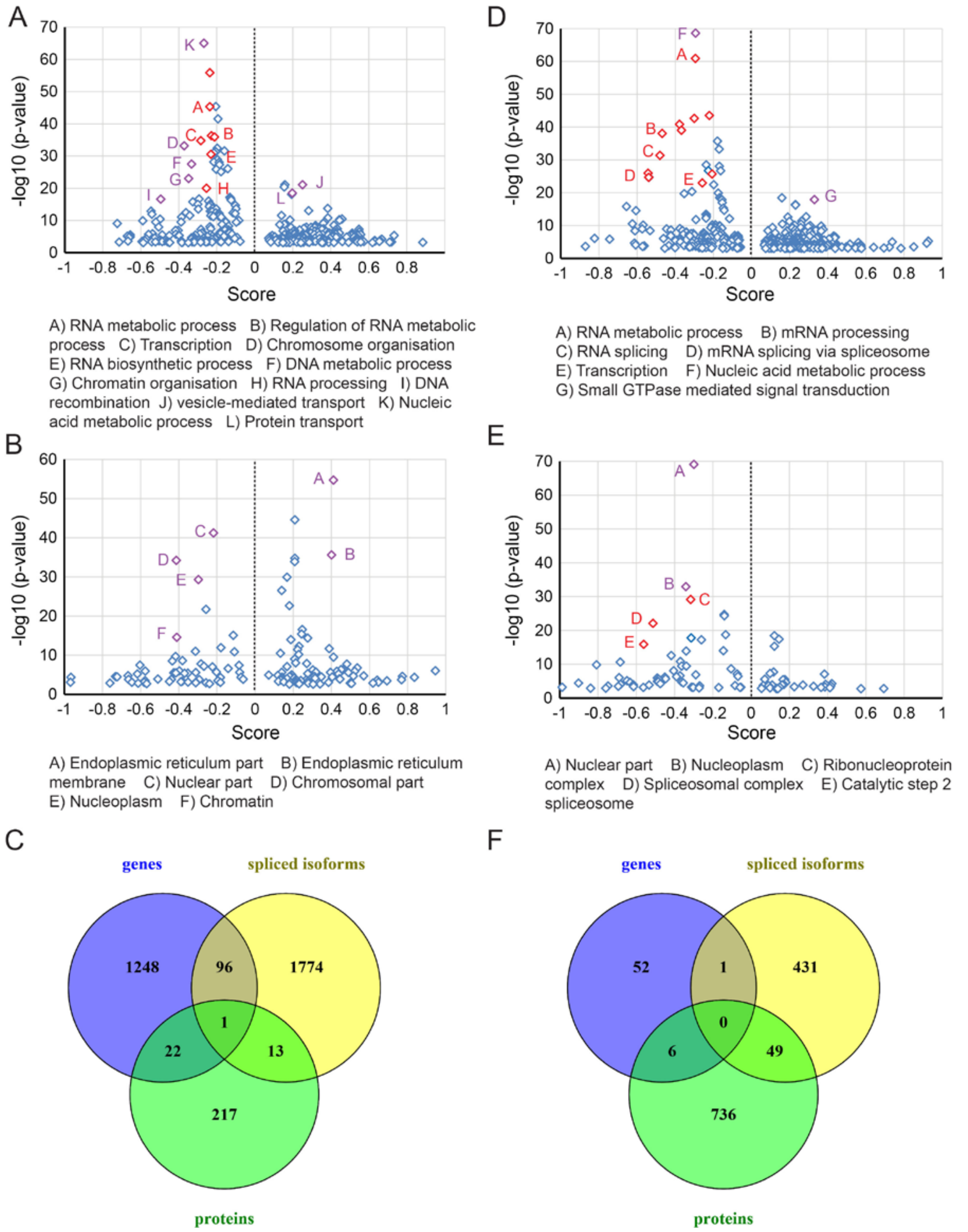


**Supplementary Figure 10. Differential protein abundance in RP11VS and CRISPR/Cas9 corrected RPE and retinal organoids. (A,B,D,E)** 1D annotation enrichment showing the most affected GO biological processes **(A,C)** and cellular components **(B,D)** in retinal organoids **(A,B)** and RPE **(D,E)**. Candidate categories that show a low or a high score and high statistical significance are highlighted in red (for RNA-related metabolic processes) or in magenta **(C,F)** The overlap between differential gene expression, alternatively spliced transcripts and protein abundance in retinal organoids **(C)** and RPE **(F)**. A Z-score cut off > 1.5 and < -1.5 was

used for the identification of differentially expressed proteins between RP11VS and Cas9-RP11VS.

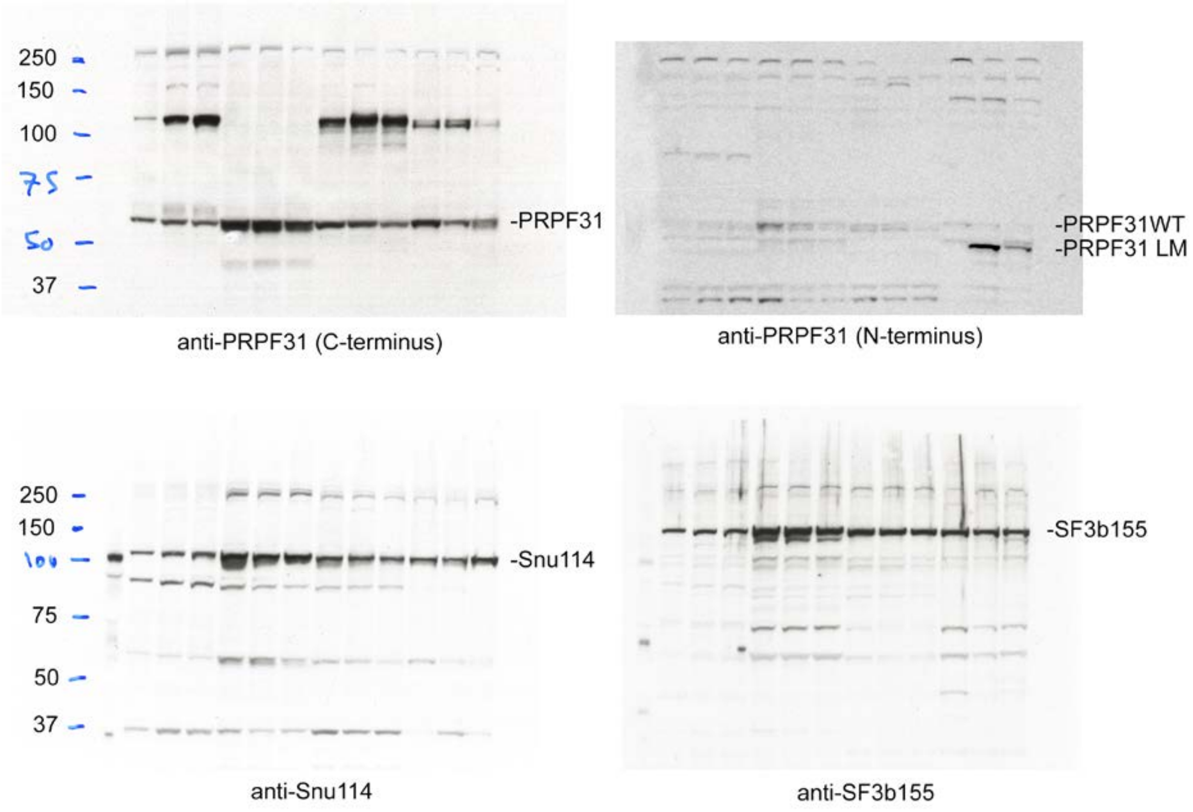


**Supplementary Figure 11.** Uncropped blots related to Figure 3C showing western immunoblotting with anti PRPF31 (C-terminus) in the top left, anti PRPF31 (N-terminus) in the top right, anti-Snu144 in the bottom and anti-SF3b155 in the bottom right panel. Sample ordering across the lanes is identical to Figure 3C. Data are representative of 3 independent repeats
