# Supplementary material for: Disrupted alternative splicing for genes implicated in splicing and ciliogenesis causes PRPF31 retinitis pigmentosa
Source: Nat Commun. 2018 Oct 12;9:4234. doi: 10.1038/s41467-018-06448-y (PMC6185938; doi:10.1038/s41467-018-06448-y)
Supplement: Supplementary file 2 — Description of Additional Supplementary Files [file 41467_2018_6448_MOESM2_ESM.pdf]

Description of Additional Supplementary Files

File Name: Supplementary Data 1
Description: Summary of clinical data for all RP11 patients and controls.

File Name: Supplementary Data 2
Description: Differential gene expression in fibroblasts, iPSCs, retinal organoids and RPE samples of all RP11 patients versus all WT controls (padj< 0.05).

File Name: Supplementary Data 3
Description: Summary of GO terms for cellular components of genes differentially expressed in fibroblasts, iPSCs, retinal organoids and RPE samples of all RP11 patients versus all WT controls

File Name: Supplementary Data 4
Description: Differential exon usage in fibroblasts, iPSCs, retinal organoids and RPE samples of all RP11 patients versus all WT controls (p< 0.05 and inclusion difference of > than 0.05).

File Name: Supplementary Data 5
Description: Summary of GO terms for cellular components of genes differentially spliced in fibroblasts, iPSCs, retinal organoids and RPE samples of all RP11 patients versus all WT controls

File Name: Supplementary Data 6
Description: A summary of sequencing data, antibodies, oligonucleotides, snRNP probes and CRISPR/Cas9 reagents.

File Name: Supplementary Data 7
Description: Off-target site sequencing analysis for gRNA (AGATCCAACCGTATGAGCTTCGG) of PCR products using corrected RP11-1VS patient genomic DNA.

File Name: Supplementary Data 8
Description: Differential protein abundance between RP11VS and Cas9-RP11VS corrected RPE cells and associated GO analysis.

File Name: Supplementary Data 9
Description: Differential protein abundance between RP11VS and Cas9-RP11VS corrected retinal organoids and associated GO analysis.
