# Supplementary material for: Disrupted alternative splicing for genes implicated in splicing and ciliogenesis causes PRPF31 retinitis pigmentosa
Source: Nat Commun. 2018 Oct 12;9:4234. doi: 10.1038/s41467-018-06448-y (PMC6185938; doi:10.1038/s41467-018-06448-y)
Supplement: Supplementary file 3 — Supplementary Data 1 [file 41467_2018_6448_MOESM3_ESM.docx]

| **Sample** | **Lab identifier** | **Age** | **Age of onset of any symptoms** | **Sex** | **Type** | **Genotype** | **Phenotype** |
| --- | --- | --- | --- | --- | --- | --- | --- |
| WT1 | SB-Ad2 | 51 | - | M | - | Wild type | Unaffected |
| WT2 | SB-Ad3 | 31 | - | F | - | Wild type | Unaffected |
| WT3 | SB-Ad4 | 68 | - | M | - | Wild type | Unaffected |
| RP11S1 | F116 | 69 | 10 | F | PRPF31 | c.1115_1125 del11 | Severe - Presented with night blindness aged 10yrs old with decreased central vision by 30yrs. Cataract requiring surgery in 50s. Current visual acuity 6/60 and 6/24. Bilateral, extensive bone spicule pigmentation, attenuated arterioles and pale optic discs with small preserved islands of RPE in macula. |
| RP11M | F118 | 52 | 10 | M | PRPF31 | c.1115_1125 del11 | Moderate - Presented with night blindness aged 10yrs old. Cataracts requiring surgery at early age. Current. Acuity 6/9 both eyes and still meeting legal requirements for driving. Ring of bone spiculation (about 4 Optic Disc Diameters wide) anterior to arcades, maculae normal. |
| RP11VS | F119 | 45 | 10 | M | PRPF31 | c.1115_1125 del11 | Very severe - Presented with night blindness aged 10yrs old with decreased central vision by 30yrs. Also has right optic nerve hypoplasia. Current visual acuity - No perception of light right eye ~~t~~ and 6/18 left. Bilateral extensive bone spicule pigmentation, attenuated arterioles and pale optic discs and visual field restricted to around 5 degrees from fixation. |
| RP11S2 | F255 | 49 | 15 | F | PRPF31 | c.522_527+10del | Severe – Presented with night blindness aged 15yrs old with decreased central vision by 30yrs. Current visual acuities 6/18 and 6/36 with a refraction of -2 and bilateral posterior subcapsular cataracts. Bilateral extensive bone spicule pigmentation, attenuated arterioles and pale optic discs. |

**Supplementary Data 1: Summary of clinical data for all RP11 patients and controls.**

The c.1115_1125del11 deletion in exon 11 of *PRPF31* NC_000019.10 (NM_015629.3) results in a long mutant (LM) NMD-insensitive mRNA transcript that causes a frame-shift after amino acid 371 leading to a truncated protein of 469 residues, and a NMD-sensitive short mutant (SM) form in which exon 11 is skipped during splicing and a premature termination codon is created in exon 12 (Rio Frio et al., 2008). The second *PRPF31* mutation NC_000019.10 (NM_015629.3):c.522_527+10del in exon 6 deletes codons 175 and 176, the last two in exon 6, encoding glutamine and glycine residues. However, it also deletes the first 10 bp of intron 6, including the exon 6/intron 6 boundary and splice donor site, thus abolishing the exon 6 splice donor site. This may give rise to an mRNA transcript which includes intron 6, adding seven novel amino acids then terminating the encoded protein, or could lead to the skipping of exon 6 (Ghazawy et al., 2007).

[Rio Frio T](https://www.ncbi.nlm.nih.gov/pubmed/?term=Rio%20Frio%20T%5BAuthor%5D&cauthor=true&cauthor_uid=18317597), [Wade NM](https://www.ncbi.nlm.nih.gov/pubmed/?term=Wade%20NM%5BAuthor%5D&cauthor=true&cauthor_uid=18317597), [Ransijn A](https://www.ncbi.nlm.nih.gov/pubmed/?term=Ransijn%20A%5BAuthor%5D&cauthor=true&cauthor_uid=18317597), [Berson EL](https://www.ncbi.nlm.nih.gov/pubmed/?term=Berson%20EL%5BAuthor%5D&cauthor=true&cauthor_uid=18317597), [Beckmann JS](https://www.ncbi.nlm.nih.gov/pubmed/?term=Beckmann%20JS%5BAuthor%5D&cauthor=true&cauthor_uid=18317597), [Rivolta C](https://www.ncbi.nlm.nih.gov/pubmed/?term=Rivolta%20C%5BAuthor%5D&cauthor=true&cauthor_uid=18317597). Premature termination codons in PRPF31 cause retinitis pigmentosa via haploinsufficiency due to nonsense-mediated mRNA decay. [J Clin Invest.](https://www.ncbi.nlm.nih.gov/pubmed/18317597) 2008 Apr;118(4):1519-31.

[S Ghazawy](https://www.ncbi.nlm.nih.gov/pubmed/?term=Ghazawy%20S%5BAuthor%5D&cauthor=true&cauthor_uid=17895420), [K Springell](https://www.ncbi.nlm.nih.gov/pubmed/?term=Springell%20K%5BAuthor%5D&cauthor=true&cauthor_uid=17895420), [V Gauba](https://www.ncbi.nlm.nih.gov/pubmed/?term=Gauba%20V%5BAuthor%5D&cauthor=true&cauthor_uid=17895420), [M A McKibbin](https://www.ncbi.nlm.nih.gov/pubmed/?term=McKibbin%20MA%5BAuthor%5D&cauthor=true&cauthor_uid=17895420), and [C F Inglehearn](https://www.ncbi.nlm.nih.gov/pubmed/?term=Inglehearn%20CF%5BAuthor%5D&cauthor=true&cauthor_uid=17895420). Dominant retinitis pigmentosa phenotype associated with a new mutation in the splicing factor PRPF31. [Br J Ophthalmol](https://www.ncbi.nlm.nih.gov/pmc/articles/PMC2000988/). 2007 Oct; 91(10): 1411–1413.
